# Optimization of the production process for the anticancer lead compound illudin M: process development in stirred tank bioreactors

**DOI:** 10.1186/s12934-022-01870-w

**Published:** 2022-07-18

**Authors:** Lillibeth Chaverra-Muñoz, Stephan Hüttel

**Affiliations:** 1 Department of Microbial Drugs, Helmholtz Centre for Infection Research, Brunswick, Germany; 2 German Centre for Infection Research (DZIF), Partner Site Hannover-Braunschweig, Brunswick, Germany

**Keywords:** Natural products, Anticancer molecules, Basidiomycota, Fungal biotechnology, Bioprocess optimization, Acetate feed, RAMOS, DASGIP

## Abstract

**Background:**

The fungal natural products illudin S and M have been investigated as precursors for the development of semisynthetic anticancer agents such as Irofulven (illudin S derivative) which is currently in phase II clinical trials. Recently, illudin M derivatives have shown improved in vitro selectivity towards cancer cells encouraging further investigation. This requires a stable supply of the precursor which is produced by Basidiomycota of the genus *Omphalotus*. We have recently reported a robust shake flask process for the production of gram quantities of illudin M from *Omphalotus nidiformis* aiming to transfer that process into stirred tank bioreactors, which can be used in a commercial production set-up. However, process transfer across different systems is not straightforward and particularly challenging when the producer is morphologically complex. There are only a few reports that address the development of bioprocesses for the production of compounds from Basidiomycota as these organisms have not been extensively studied because of their complex life cycles and often are difficult to cultivate under laboratory conditions.

**Results:**

The recently developed shake flask process delivering stable titers of ~ 940 mg L^−1^ of illudin M was investigated using off-gas analysis to identify critical parameters which facilitated the transfer from shaken into stirred tank bioreactors. Comparable titers to the shake flask process were achieved in 2 L stirred tank bioreactors (1.5 L working volume) by controlling growth of biomass with a carefully timed pH-shift combined with an improved precursor-feeding strategy. A scale-up experiment in a 15 L bioreactor (10 L working volume), resembling the process at 1.5 L resulted in 523 mg L^−1^ and is the starting point for optimization of the identified parameters at that scale.

**Conclusion:**

By identifying and controlling key process parameters, the production process for illudin M was transferred from shake flasks into 2 L stirred tank bioreactors reaching a comparable titer (> 900 mg L^−1^), which is significantly higher than any previously reported. The insights obtained from 10 L scale pave the way towards further scale-up studies that will enable a sustainable supply of illudin M to support preclinical and clinical development programs.

**Supplementary Information:**

The online version contains supplementary material available at 10.1186/s12934-022-01870-w.

## Background

Natural products have traditionally been the most important source of molecules for pharmaceutical applications. However, the development of lead compounds towards clinical use, requires extensive studies utilizing significant quantities of such molecules which typically are difficult to obtain from their natural sources [1]. Illudins are an important class of highly cytotoxic sesquiterpenes produced by Basidiomycota. In the past decades they have been investigated as precursors for the production of semisynthetic derivatives with potent anticancer properties [2, 3], and Irofulven—a derivative of illudin S—is currently in phase II clinical trials for the treatment of prostate cancer [4]. Recently, semisynthetic derivatives of illudin M have shown improved selectivity towards malignant cells, but the current limited availability of the compound hampers further studies [5–7]. We therefore aimed to develop a biotechnological process, enabling a stable illudin M supply using the natural producer *Omphalotus nidiformis*. The first step was the improvement of product titers by developing an optimized fed-batch process in shake flasks, which could be suitable for subsequent process transfer into a scalable system such as stirred tank bioreactors [8].

Submerged cultivation in stirred tank bioreactors is the preferred production method of fungal secondary metabolites since this enables precise measurement and strict control of critical process parameters. These are prerequisites to ensure consistent quality of materials used for pharmaceutical purposes [9]. The widespread use of stirred tank bioreactors and the acceptance by regulatory authorities restricts the development of alternative procedures. However, filamentous organisms usually exhibit complex morphologies in liquid cultures which can influence the hydrodynamics in the bioreactor and factors such as media composition and mixing can have direct impact on the physiology and morphology of the producer. Such interdependencies between process parameters and the properties of filamentous organisms are often poorly understood and unpredictable, which makes process development and scale-up very challenging tasks [10–12].

Shear and hydromechanical stress have been considered important factors that can strongly influence morphology during submerged cultivation of filamentous organisms and therefore parameters such as volumetric power input and the energy dissipation circulation function (EDCF) are commonly used as scale up criteria [13–15]. However, scale up is considered a multidimensional problem and there is no single way to approach it since interactions between process parameters will also depend on the biological characteristics of the producer organism [16]. The increasing availability of novel tools and strategies such as image analysis, genomic technologies together with morphology and genetic engineering, have contributed to the development of fungal biotechnology and the understanding of the characteristics of filamentous organisms in submerged cultures [17–21]. Most reports however relate to products from Ascomycota or dealing with engineered yeasts as heterologous producers. Reports relating to compounds obtained from Basidiomycota are limited; particularly descriptions concerning the production of pharmaceuticals from these organisms [22–24].

We describe one approach that transfers the production of illudin M from a high titer shake flask process into a scalable process in stirred tank bioreactors. We show that the identification of key performance parameters in shake flask experiments helped to define critical factors for the successful transfer to the scalable system. Illudin M titers of > 900 mg L^−1^ were achieved in 1.5 L cultivations in stirred tank bioreactors. The optimized parameter setup was transferred to a 10 L process in a stainless steel bioreactor that delivered ~ 500 mg L^−1^ of illudin M; this concentration is significantly higher than any previously reported using stirred tank bioreactors. This is the second of a series of three reports addressing improvements of the biotechnological production of illudin M [8, 25]. The aim of this work was the transfer of the optimized shake flask process into stirred tank bioreactors and set the base for further scale-up development ensuring a stable supply of illudin M for further pre-clinical and clinical studies.

## Results

### Process characterization in monitored shake flasks

To identify critical process parameters, the optimized shake flask process for the production of illudin M [8] was performed using the online Respiration Activity Monitoring System RAMOS® [26]. By setting the aeration rate to 4.7 mL min^−1^ in the RAMOS flasks it was possible to resemble the hydrodynamics and gas composition of the conventional 500 mL Erlenmeyer flasks with membrane caps. The carbon dioxide transfer rate (CTR) and oxygen transfer rate (OTR) were recorded over the course of the cultivations. Two experiments were performed in duplicates (batch and fed-batch cultivations) in order to investigate the influence of the acetate and glucose feed on the respiratory activity of the producer organism.

**Fig. 1:**
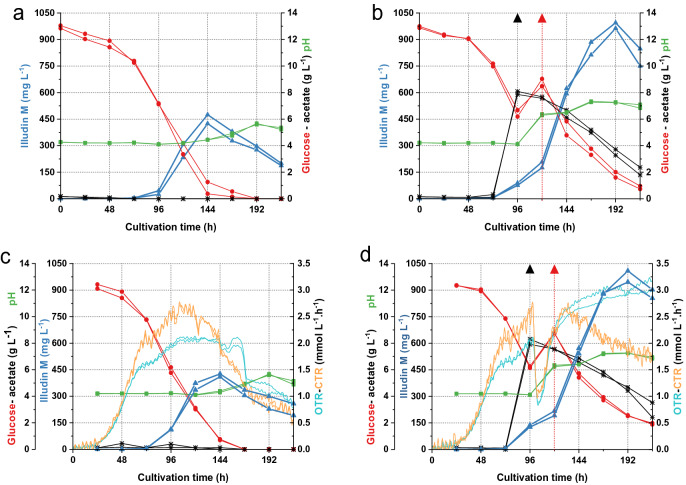
Process kinetics from cultivations in standard shake flasks and RAMOS flasks for production of illudin M with *O. nidiformis*. Experiments in conventional Erlenmeyer flasks: **a** batch process used as control, **b** optimized fed-batch process. Experiments in RAMOS flasks: **c** batch process used as control, **d** optimized fed-batch process. The fed-batch cultivations were fed with acetate (8 g L^−1^) at 96 h indicated with a black triangle and glucose (6 g L^−1^) at 120 h indicated with a red triangle. All curves illustrating the course of different process parameters are colored according to the color of the axis labels. The highest measured concentration of illudin M in the batch cultivations was ~ 420 mg L^−1^ at 144 h and in the fed-batch ~ 1000 mg L^−1^ at 192 h. Illudin M titers were derived from cell free culture supernatant

**Fig. 2:**
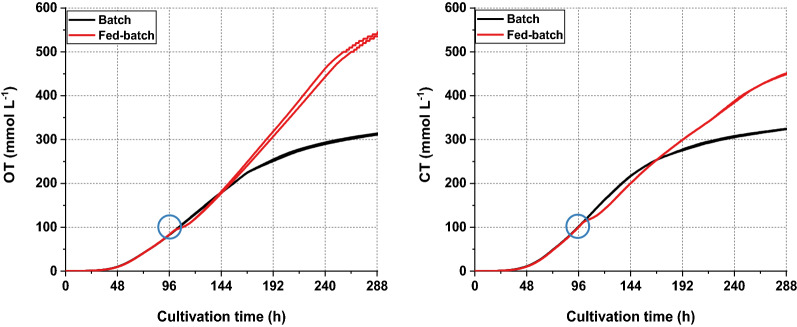
Comparison of CO_2_ production on O_2_ consumption in batch and fed-batch cultivations. The curves indicate the OT andCT obtained from duplicate experiments. The red curves illustrate the fed-batch data and the black curves illustrate the data from the batch cultivation indicating a lower O_2_ consumption and CO_2_ production in the batch process. The blue circle highlights the time of feeding

The process kinetics for product formation, substrate consumption and pH of the RAMOS cultivations were identical to those obtained from the conventional shake flasks as shown in Fig. 1. The titers of illudin M shown in the kinetics of all the cultivations during this work are the concentration of product measured in cell free supernatant as biomass was removed by centrifugation prior to the extraction of the samples. Biomass volume never exceeded 6% of the total volume of the culture in the case of the shake flask and 10% in the case of bioreactor cultivations. Figure 1c, d illustrate the processes performed in the RAMOS system with the respective courses of CTR and OTR during the experiments. Both processes showed an exponential increase of the OTR and CTR values between 24 and 72 h, which changed to a slower increase until 96 h reaching a plateau in the case of the OTR values of the batch process. It was also evident that the feed of potassium acetate and/or the associated increase of pH at 96 h caused an instant decrease of both the OTR and CTR values in the fed-batch process, indicating reduced respiratory activity that recovered within 24 h (Fig. 1d). While the CTR and OTR values decreased sharply after 144 h in the batch cultivation, the OTR in the fed batch process increased up until 216 h and the CTR decreased to a lesser extent than in the batch cultivation. The differences in the respiratory activity between both processes were quantified by plotting an overlay of the integrated OTR and CTR values (OT and CT) as shown in Fig. 2, which indicated a higher and prolonged respiratory activity in the fed-batch process.

It is evident that the feed of acetate and glucose influenced the metabolism and respiratory activity of *O. nidiformis* which was beneficial for improved illudin M titers. We hypothesized that by achieving a similar process profile in stirred tank bioreactors, comparable titers to the shake flask process would be achieved. We recently reported that the feed of acetate and/or the associated increase in pH, reduced the growth of the producer organism and concluded that an optimal quantity of active biomass during the production phase was crucial for improved product titers [8].

### Evaluation of pH as process parameter for biomass control in stirred tank bioreactors

**Fig. 3:**
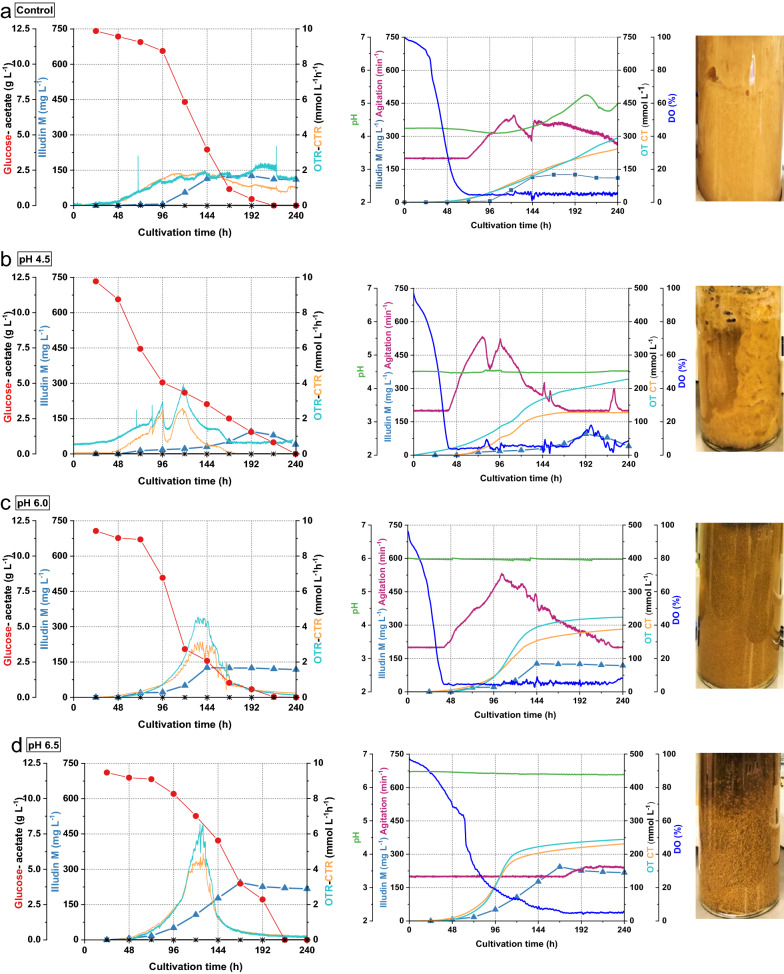
Process kinetics from uncontrolled and pH controlled cultivations in stirred tanks (1.5 L) and culture appearance at harvest time. All cultures were prepared with *O. nidiformis* cultivated in G13.5/C7 medium at 23 °C, aeration 0.3 vvm (27 sL h^−1^) and dissolved oxygen (DO) was maintained at 5% by increasing the stirring speed. **a** No pH control **b** pH 4.5 **c** pH 6.0 and **d** pH6.5. All curves illustrating the course of different process parameters are colored according to the color of the axis labels. Illudin M titers were derived from cell free culture supernatant. The concentration of wet biomass at harvest was as follows for **a** 45.83 g L^−1^; **b** 40.81 g L^−1^; **c** 23.59 g L^−1^ and **c** 18.07 g L^−1^

To confirm that pH per se had an effect on biomass formation we performed a set of four batch cultivations in 2 L stirred tanks (1.5 L working volume) using the parallel fermentation system DASGIP®. The pH values of the cultures were controlled from beginning of the cultivation at pH 4.5; pH 6.0 and pH 6.5 and one experiment without pH control resembling the shake flask process was conducted for comparison. All other process parameters were fixed, and no acetate was fed. Figure 3 illustrates the process kinetics and appearance of the cultures. By looking at the appearance of the cultures it is evident that pH had a major effect on biomass formation and morphology as increasing pH values resulted in reduced biomass and more distinct pellets (less filamentous appearance) compared to the cultures at lower pH. The highest illudin M titer (243 mg L^−1^) was measured in the culture with pH 6.5 which was the culture with the lowest quantity of biomass. In the culture at pH 4.5 (Fig. 3a) we observed zones of poor mixing between the Rushton impellers due to high quantities of biomass, which was also seen in the control experiment (Fig. 3b). Comparing the OTR and CTR curves of all cultures it is obvious that in the cultivations with higher pH, the maximal values of these parameters where higher, showing a distinct pattern of sharper exponential increase and decrease of those values, while at lower pH values those kinetics were stretched over a longer time span and less pronounced.

We concluded that lower pH values promoted biomass growth while higher pH values led to reduced amounts of biomass with a different morphology and higher productivity, thus pH could be used to control biomass formation and improve product titers. We proposed that for the process in the bioreactor an initial growth phase at low pH and subsequent increase in pH to control growth and morphology, could be a strategy to achieve optimal quantities of active biomass during the production phase. Since in the shake flask process the feed of acetate (8 g L^−1^) at 96 h was crucial for achieving high titers of illudin M, we aimed to implement the same feeding strategy in stirred tank cultivations. However, the bioavailability of acetate is pH-dependent (see Additional file 1: Fig. S1), and high concentrations of undissociated acetate affect the growth of the organism and thus the production pattern. We have previously reported an inhibitory effect on *O. nidiformis* by feeding acetate and maintaining cultures at pH 4.2 in shake flask experiments [8] and hence, we considered the potential toxicity of acetate if pH changes are used for growth control in the bioreactor. For the cultivation in stirred tank bioreactors, we investigated the lowest pH value of the culture, which could cause growth inhibition when applying identical feeding conditions as in the optimized shake flask process. Since the feed of acetate at pH 6.2 in the shake flask process did not severely inhibit the strain, a lower pH was tested.

**Fig. 4:**
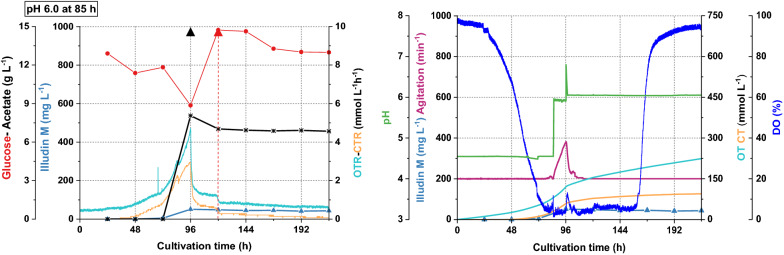
Process kinetics of a 1.5 L cultivation in a stirred tank where pH was shifted from pH 4.5 to pH 6.0 prior acetate feeding. The culture was prepared with *O. nidiformis* cultivated in G13.5/C7 medium at 23 °C, aeration 0.3 vvm (27 sL h^−1^) and dissolved oxygen (DO) was maintained at 5% by increasing the stirring speed. The pH was shifted to pH 6.0 at 85; acetate (8 g L^−1^) was fed at 96 h indicated with a black triangle and glucose (6 g L^−1^) was fed at 120 h indicated with a red triangle. All curves illustrating the course of the different process parameters are colored according to the colors of the axis labels. The sharp decrease of OTR and CTR after 96 h and the low productivity indicated inhibitory effects of the concentration of undissociated acetate at pH 6.0 under the applied conditions. Illudin M titers were derived from cell free culture supernatant

We conducted a feeding experiment in a stirred tank controlling pH at 4.5 during the growth phase and increasing to pH 6.0 (85 h) prior the acetate feeding at 96 h (8 g L^−1^). Glucose was fed at 120 h (6 g L^−1^). After the acetate feed the oxygen consumption decreased immediately (see Fig. 4) causing a reduction in stirring rate and a sharp drop of CTR and OTR. The growth of the strain seemed to be significantly affected leading to reduced production of illudin M. These results suggested that at pH 6.0 the concentration of undissociated acetate, was too high to be tolerated by the strain. We concluded that by setting pH values greater or equal to 6.2 prior to the acetate feed, growth inhibition could be circumvented.

Based on these observations we concluded that the effect of the acetate feed on biomass growth was, as previously assumed, a function of pH and an inhibition on growth by undissociated acetate at certain concentrations. This imposes two contrary effects on *O. nidiformis* when acetate (8 g L^−1^) is fed: high pH per se (> 6) can affect biomass growth as reported before (see Figs. 3, 4) and low pH (≤ 6) can reduce growth due to inhibitory effects of increasing concentrations of undissociated acetate. This indicated that careful balancing of both effects was required, which we aimed to control by dividing the process into three phases:

1. Growth phase at a low pH (4.2–4.5) for initial biomass formation
2. Biomass and morphology control phase by shifting the pH to a value higher than pH 6.2
3. The pH-controlled production phase with acetate and glucose feeding to improve product titers, resembling the conditions in the shake flask process.

### Influence of pH-shift time and pH-value on illudin M titers in stirred tank bioreactors

Since the increase of pH value should be performed prior to the acetate feed to control biomass formation, we investigated the influence of the shift time on product titers. We performed four experiments in stirred tank bioreactors (1.5 L) increasing the pH from 4.5 to 6.5 at 24 h; 48 h; 72 h and 96 h. Acetate (8 g L^−1^) was fed at 96 h and glucose (6 g L^−1^) at 120 h. Due to the high number of experiments in the following sections, the datasets are only discussed here and the complete process kinetics are provided as supplementary information.

**Fig. 5:**
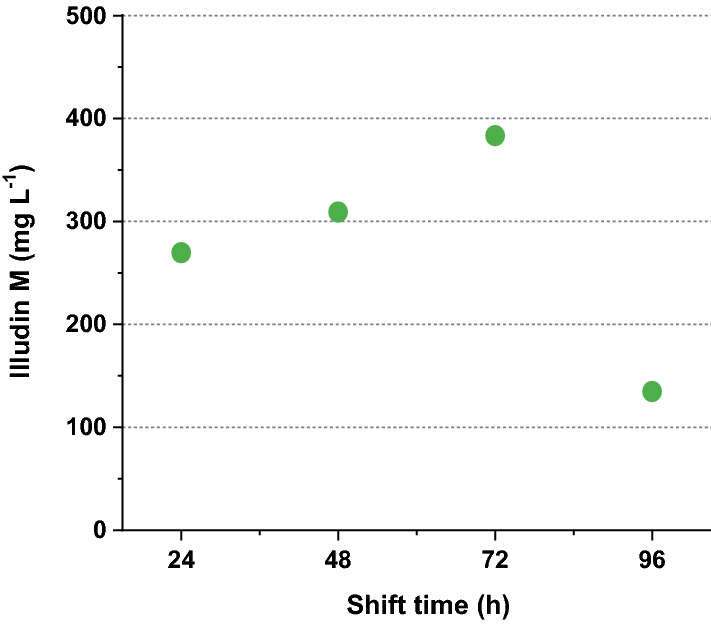
Comparison of highest product titers measured from stirred tank cultivations (1.5 L) where pH was shifted from pH 4.5 to pH 6.5 at different cultivation times. The scatter plot illustrates the concentrations of illudin M plotted against shift time, at the time of maximum productivity that in all cases occurred at 192 h regardless acetate availability or depletion. Illudin M titers were derived from cell free culture supernatant

By comparing the process kinetics (see Additional file 1: Fig. S2), the pH shift at 24 h (Additional file 1: Fig. S2a) and 48 h (Additional file 1: Fig. S2b) led to slower growth reflected by a slower decrease of the DO, slower increase of CTR and OTR and slower acetate uptake compared with shifts at 72 h and 96 h (see Additional file 1: Fig. S2c, d). The highest illudin M titer (373 mg L^−1^) was measured in the 72 h shift-experiment (see Fig. 5 and Additional file 1: Fig. S2c), which had significantly higher CTR and OTR around 144 h than in experiments in which the shifts were performed at earlier time points. This finding is consistent with the results of the pH-controlled cultivations (see Fig. 3) in which the highest product titer was measured in the culture with higher CTR and OTR values around 144 h,. The acetate uptake in this culture (see Additional file 1: Fig. S2c) was the highest within this set of experiments and considerably higher than in the shake-flask process. This led to acetate depletion at 168 h, which was probably the cause of stagnation of illudin M production and a sharp drop of OTR and CTR at that time point. A comparison of the highest titers per experiment is shown in Fig. 5.

The pattern of a sudden decrease of CTR and OTR values at lower acetate concentrations was also observed in the culture in which the pH was shifted by the time of feeding at 96 h (see additional file 1: Fig. S2d), but titers were significantly lower. These results showed that the timing of the pH shift influenced product formation and that the acetate limitation in the production phase must be compensated by increasing the acetate feed.

In the next set of six experiments we investigated different pH shift times at 63 h; 72 h and 81 h and pH values shifting from 4.5 to 6.5 or to 6.8. To evaluate a new acetate feeding profile, the precursor was fed at 96 h (8 g L−1), 168 h (4 g L^−1^) and 192 h (4 g L^−1^). A feed of glucose was performed at 120 h (6 g L^−1^) as in the shake flask process. When comparing the process kinetics it became evident that in cultures shifted from pH 4.5 to 6.5 (see Additional file 1: Fig. S3 a, c ,e) the CTR and OTR values were higher compared to the cultures with a pH shift to 6.8 (see Additional file 1: Fig. S3b, d, f). This could result from a bigger impact of the pH 6.8 on the cell growth prior to the acetate feed, lower acetate availability at that pH, or a combination of both effects. The highest illudin M titer (753 mg L^−1^) was measured in the culture that had a pH shift to 6.5 at 81 h (see Additional file 1 Fig. S3e).

**Fig. 6:**
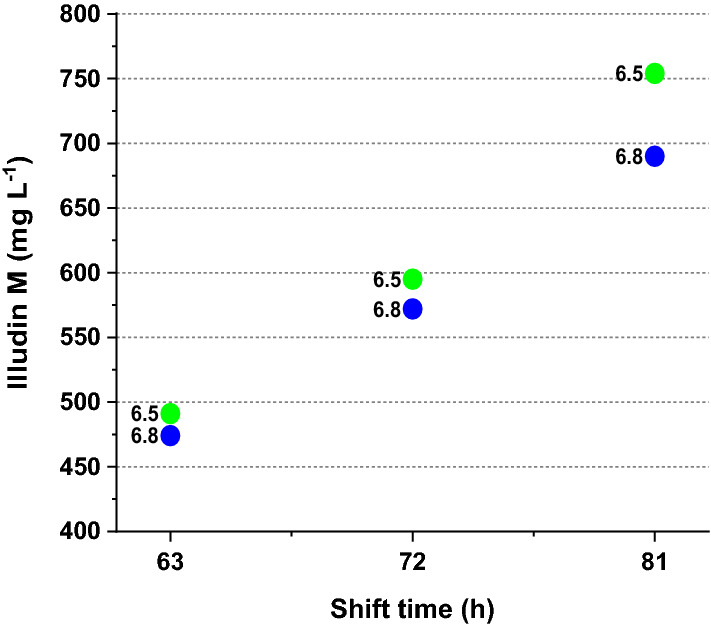
Comparison of highest product titers measured in stirred tank cultivations (1.5 L) where pH was shifted from pH 4.5 to pH 6.5 or to pH 6.8 at different cultivation times. The scatter plot illustrates the concentrations of illudin M at the time of maximum productivity (192 h) plotted against the shift time. The green and blue dots indicate the shift to pH 6.5 and pH 6.8 respectively as labelled in the plot. Illudin M titers were derived from cell free culture supernatant

A summary of the highest product titers achieved in this experiment is shown in Fig. 6. By plotting the highest product titers over shift-time it became obvious that the latter had a greater impact than the pH values investigated. In all cases earlier shift time correlated with lower titers regardless the pH.

These results indicated that the optimal shift value would be around pH 6.5, whereas the optimal shift time would be around 81 h since 96 h (Fig. 5) and pH 6.8 (Fig. 6) resulted in lower illudin M titers. Despite additional feeding of acetate at 168 h the sharp reduction of OTR and CTR around 144 h indicated a potential acetate limitation as in the previous experiment. Although the CTR and OTR values increased after the second feed at 168 h the product titers did not considerably increase after that. We also observed that the consumption of glucose was reduced in all cultures in comparison with the consumption in the shake flask process.

This critical decrease in the acetate concentration at 144 h could be observed in most of the cultures (see Additional file 1: Fig. S3a–f) suggesting that the second and third feed of acetate should be performed prior to 144 h in order to avoid acetate depletion in the production phase.

The product titers achieved in stirred tanks (4 of 6 experiments delivered > 500 mg L^−1^ of illudin M) indicated that the strategy of a timed pH-shift prior to the feeding was suitable for improving product titers in the bioreactor and that the influence of the inherent differences between shaken and stirred cultivation systems could be overcome with an adapted cultivation strategy.

### Screening of pH-value and pH-shift time for improved illudin M titers in stirred tank bioreactors

We aimed to find an optimal time point and pH value for the pH shift in stirred tank cultivations in order to reach illudin M titers comparable to the shake flaks process. In the final set of experiments we adapted the feeding strategy again to avoid acetate limitation at 144 h. The feeding of acetate was performed in all cases at 96 h (8 g L^−1^); 120 h (4 g L^−1^) and 144 h (4 g L^−1^). A feed of glucose was performed at 120 h (6 g L^−1^). By changing the feeding time we expected to close the “acetate feeding gap”, achieve titers comparable to the shake flaks process and being able to predict the best operating range of our control parameters. Based on the previous experiments we expected the optimum shift time around 81 h and pH 6.5. We investigated a broader range of values around the expected maximum to create a data set covering the hypothetical optimum and allowing the generation of a mathematical model of the dependency of the illudin M titer on the two independent variables pH-value and pH-shift time. Since the number of experiments we could carry out at once was limited, two experimental subsets were planned as listed in Table 1, which presents the experimental design.

**Table 1:** Experimental design matrix describing illudin M production in response to timed pH change

Subset	Trial	Factor 1 (x1) Shift Time (h)	Factor 2 (x2) pH	Response	
				Illudin M (mg L^−1^)	
				Actual	Predicted
1	1	78.0	6.50	698	888
1	2	85.0	6.00	43	132
1	3	85.0	6.50	995	981
1	4	85.0	6.25	901	708
1	5	85.0	7.00	605	621
2	6	74.0	6.20	682	606
2	7	74.0	6.50	736	756
2	8	78.5	6.80	797	703
2	9	83.0	6.20	608	647
2	10	87.5	6.80	953	950
2	11	92.0	6.20	421	399
2	12	92.0	6.50	851	900

The first subset consisted of five experiments. The run at pH 6.00 and pH 7.00 were to confirm the pH boundaries in which growth and production would be either inhibited by high acetate availability or by high pH value; they were performed once since there was no significant production expected based on the outcome of the previous experiments.

The second subset consisted of seven experiments designed to cover pH values and shift time around the expected optimum, which complemented the first subset in order to calculate a quadratic model from the complete dataset (full kinetics are shown in Additional file 1: Fig. S4). These data were used to build a second order model using lm (stats version 3.6.2) in R (version 4.1.1). The performance of the model is represented by Eq. 1:

1 $${\text{c}}_{\text{illudin M}}=-81533916-117.697x1+26428.789x2+64.906x1x2-1.784x1^{2}-2419.782x2^{2}$$

With a stationary point (maximum) at pH shift time 87.64 h and pH 6.64. Residual standard error: 129.9 on 6 degrees of freedom (DF), Multiple R^2^: 0.8653, Adjusted R^2^: 0.753 and F-statistic: 7.707 on 5 and 6 DF, p-value: 0.01368.

The expected drop in production at pH < 6.2 and pH > 7.0 led to an unconventional coverage of the experimental space considering standard experimental designs but was considered suitable for the purpose of estimating the conditions of maximum response of the dependent variable, which has been narrowed down in the initial shake flask experiments were expected maximum titers of ~ 1000 mg L^−1^ had been evaluated. Experimental data was analyzed by ANOVA using R (version 4.1.1), the results are shown in Table 2.

**Table 2:** Analysis of variance (ANOVA) for illudin M production in response to pH values and pH-shift time

	df	SS	MS	F value	Pr > F
x1	1	2917	2917	0.1729	0.692014
x2	1	201,936	201,936	11.9708	0.013469
x1^2^	1	5842	5842	0.3463	0.577659
x2^2^	1	373,285	373,285	22.1283	0.003312
x1: x2	1	66,045	66,045	3.9152	0.095198
Residuals	6	101,214	16,869		

df: degree of freedom; SS: sum square; MS: mean square; Pr > F probability value

**Fig. 7:**
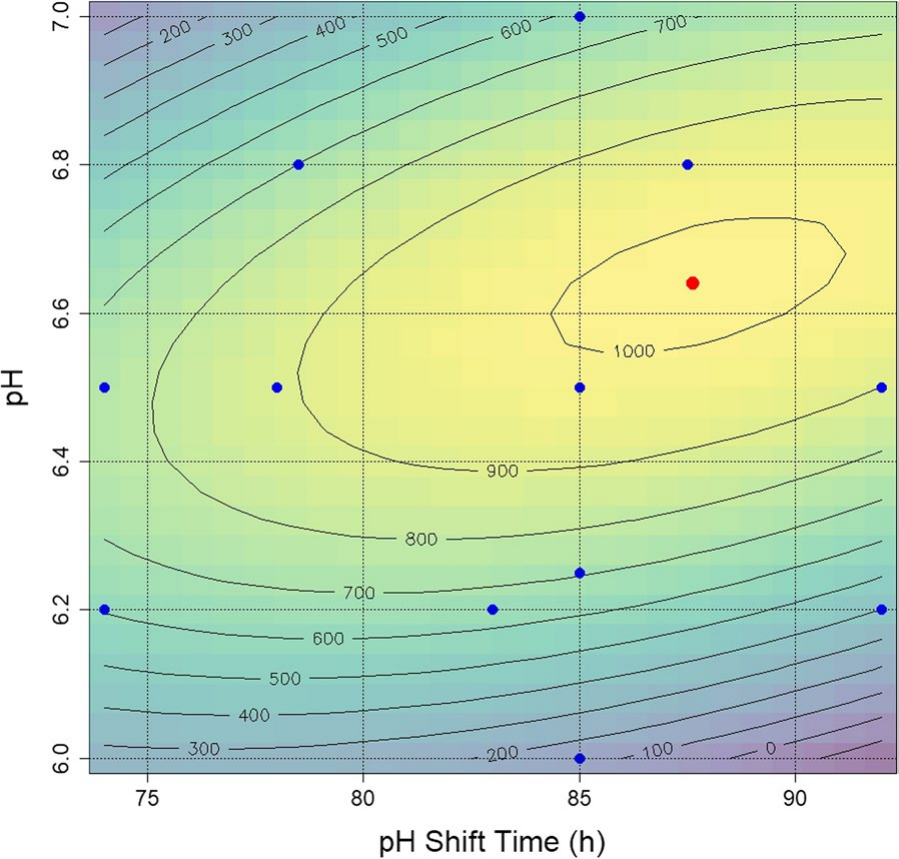
Contour plot derived from experimental data illustrating the influence of different pH values and corresponding shift time on illudin M titers. Background colors from blue to yellow indicate increasing illudin M titers, underlined by grey contour lines derived from the second order model built in R with the lm library. Model summary: Multiple R^2^: 0.8653, Adjusted R^2^: 0.753 and F-statistic: 7.707 on 5 and 6 DF, p-value: 0.01368. A maximum was calculated at shift time: 87.64 h and pH 6.64. Blue dots indicate the pH and shift time evaluated to generate the lm model in R. The red dot highlights the parameter range of the expected maximum for illudin M production

To illustrate the effect of the independent variables pH shift time and pH shift value on the dependent variable (illudin M titer), the second order model was plotted as 2D contour graph (Fig. 7). The experimental combinations analyzed to establish the model are indicated in blue and the calculated maximum is indicated in red.

The culture in which the pH was shifted to 6.50 at 85 h reached the highest illudin M titer as illustrated in Additional file 1: Fig. S4d (995 mg L^−1^ at 192 h). These results show that the shift of the pH at a specific time and to a certain value influenced the process performance resulting in a condition of the culture in which product titers were improved by the adapted feeding of precursors, ultimately achieving titers comparable to the shake flask process.

**Fig. 8:**
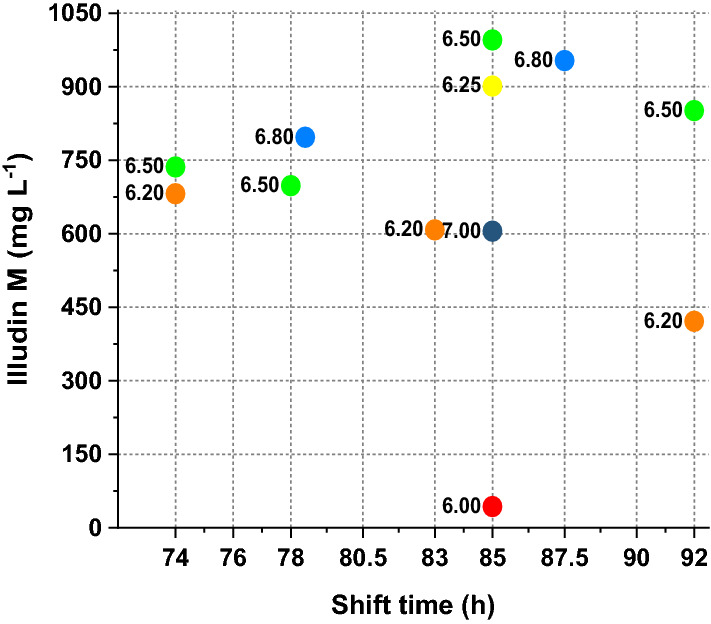
Comparison of final product titers measured from stirred tank cultivations during screening of optimal pH shift time and final value. The scatter plot illustrates the concentrations of illudin M at the time of maximum productivity. Dots are colored according to the pH evaluated, the labels indicate the pH value after the shift. Experiments where pH was shifted at 74 h reached the maximum titers at 168 h and all other experiments at 192 h. Illudin M titers were derived from cell free culture supernatant

With the exception of the cultures with pH-shift time 74 h, in all these experiments the highest titers were measured around 192 h despite acetate and glucose availability or limitation, or differences in culture pH and pH-shift time. The comparison of the highest titers of all experiments is shown in Fig. 8 where titers are correlated with the shift times and plotted together with the corresponding pH values. Full datasets are shown in supplementary information (see Additional file 1: Fig. S4).

The outcome of this experiment defined the parameter range in small-scale stirred tank reactors that resulted in titers comparable to the shake flask process. In summary, 10 out of 12 experiments reached high illudin M titers (> 600 mg L^−1^) including 3 experiments around the expected optimum that reached > 900 mg L^−1^ of illudin M.

### Analysis of process profiles: shake flasks vs 2 L stirred tanks

**Fig. 9:**
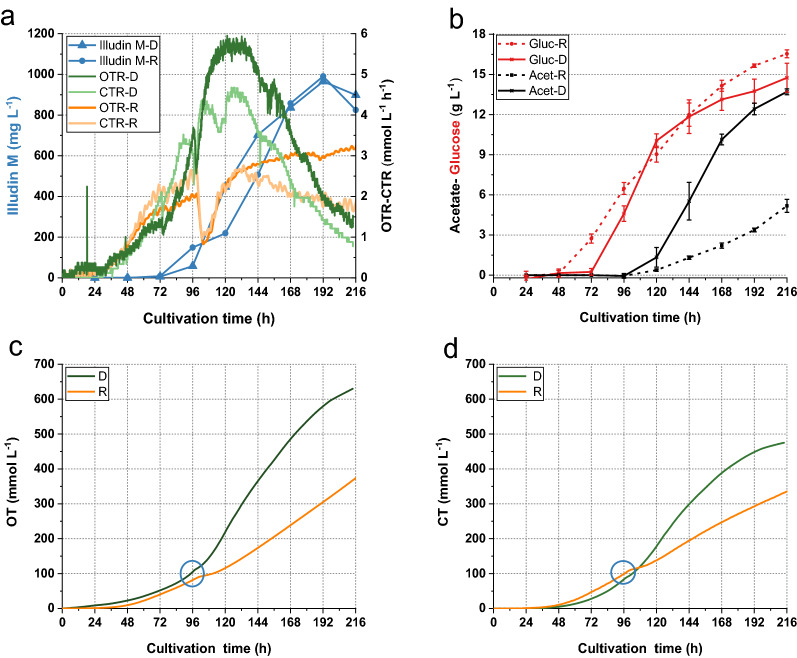
Comparison of process kinetics from optimized cultivations performed in shake flaks RAMOS (200 mL) and stirred tank DASGIP (1.5 L). Legends marked with D illustrate process values from the DASGIP cultivation and R from RAMOS cultivation. **a** Overlay of OTR-CTR and product formation over the cultivation time of the DASGIP cultivation with highest product titers and a RAMOS cultivation. **b** Total amount of glucose and acetate consumed over the cultivation time calculated from the difference between initial concentrations and the measured concentration in the daily samples. Dashed curves illustrate the data from RAMOS cultivations and solid curves illustrate the DASGIP cultivations. Error bars indicate one standard deviation (1SD) of the mean with n = 4 for RAMOS and n = 3 for DASGIP cultivations. **c**, **d** Illustrate a comparison of averages of the integrated OTR and CTR values (OT-CT) (RAMOS n = 4 and DASGIP n = 3). The blue circle highlights the time of the first acetate feed (96 h)

To compare the performance of the processes during the cultivation time an overlay of the process kinetics from RAMOS (200 mL) and DASGIP (1.5 L) cultivations is illustrated in Fig. 9. The comparison of substrates kinetics (see Fig. 9b) showed that the glucose consumption was similar in both processes while the acetate consumption in the stirred tank was significantly higher than in the shake flask process. By comparing the OTR and CTR curves (Fig. 9a) it was evident that both processes showed a similar pattern during the first 96 h. After the acetate feed the respiratory activity in the stirred tank was much higher compared to the shake flask process. This was also reflected by the evaluation of the of the OT and CT values of both cultivation types (see Fig. 9c, d). From this comparison it is evident that at the end of the batch phase, by the time of acetate feeding, both processes had a comparable respiratory and metabolic activity despite the differences between both systems. Product formation curves were slightly different between the systems but highest product titers occurred at the same time point.

### Scale up experiment: from 1.5 L to 10 L scale

One scale-up experiment was performed, in order to establish if the process with highest titers in 1.5 L cultivations was transferable to 10 L scale without significant decrease in illudin M production. The initial stirring speed was set to 150 min^−1^ to match the initial tip speed of the small stirred tank. Acetate was fed at 96 h (8 g L^−1^), 120 h (4 g L^−1^) and 144 h (4 g L^−1^). A feed of glucose was performed at 120 h (6 g L^−1^).

**Fig. 10:**
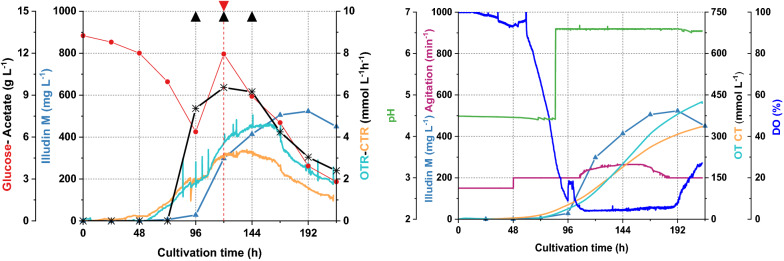
Process kinetics of a 10 L stirred tank cultivation where pH was shifted from pH 4.5 to pH 6.5 at 85 h. The culture was prepared with *O. nidiformis* cultivated in G13.5/C7 medium at 23 °C, 0.3 vvm (180 sL h^−1^) aeration and dissolved oxygen (DO) was maintained at 5% by increasing the agitation speed. Acetate was fed at 96 h (8 g L^−1^), 120 h (4 g L^−1^) and 144 h (4 g L^−1^) indicated with black triangles. A feed of glucose was performed at 120 h (6 g L^−1^) indicated with a red triangle. The plots illustrate two sets of data with the full kinetics of the experiment. All curves illustrating the course of the different process parameters are colored according to the colors of the axis labels. Illudin M titers were derived from cell free culture supernatant

The process kinetics are illustrated in Fig. 10 and showed a similar pattern as expected from the small scale process. After careful visual inspection of the culture, the initial stirring speed was increased to 200 min^−1^ at 48 h since 150 min^−1^ seemed to be too low resulting in poor mixing and settling of particles in non-turbulent zones (observable through the sight glass). This increase in stirring speed led to increased DO values after 48 h, followed by a steep drop of the DO from 72 h (see Fig. 10).

**Fig. 11:**
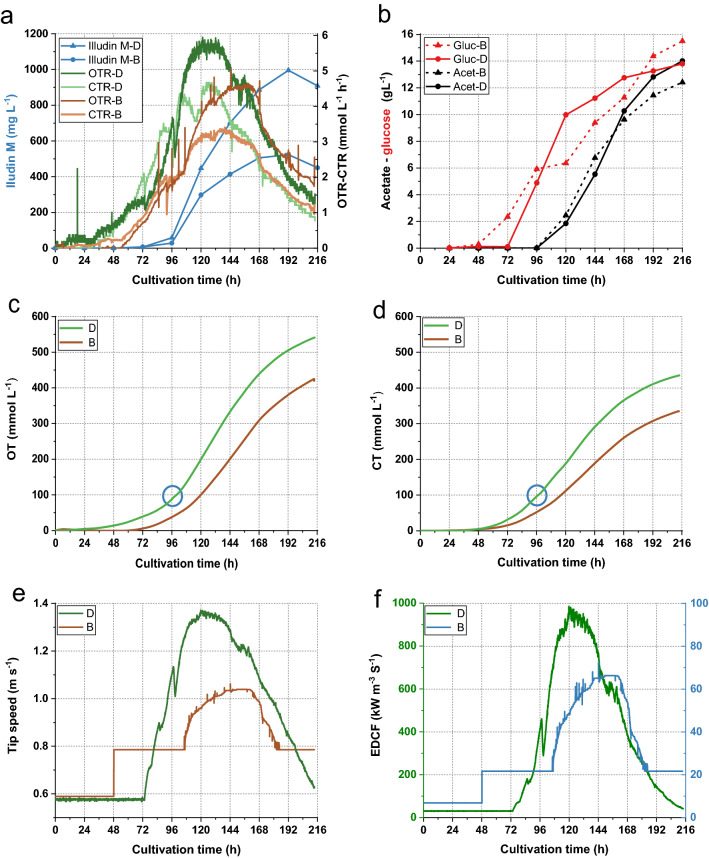
Comparison of process kinetics from cultivations of 1.5 L (DASGIP) and 10 L (BBI) in stirred tank bioreactors. Legends marked with D illustrate process values from DASGIP cultivation and B from BBI cultivation. **a** Kinetics of OTR-CTR and product formation over the cultivation time. **b** Consumption of glucose and acetate over the cultivation time. Dashed curves illustrate the data from BBI cultivation and solid curves the DASGIP cultivation **c**, **d** Illustrate a comparison of the integrated OTR and CTR values (OT and CT) and the blue circle highlights the time for acetate feeding (96 h). **e** Comparison of tip speed. **f** Comparison of the approximated EDCF values calculated with energy dissipation circulation function. Stirring speed was increased from 150 min^−1^ to 200 min^−1^ at 48 h in the 10 L cultivation (**e**, **f**), which explains the step-wise increase of the values at that time point

The highest titer measured in 10 L scale was 523 mg L^−1^ which was lower than the highest measured in the small scale process. Figure 11 illustrates an overlay of the process kinetics from the 1.5 L and the 10 L cultivations. Comparing the consumption of substrates no major differences were observed (see Fig. 11b). The comparison of OTR and CTR values (see Fig. 11a) indicated that the kinetics of the 10 L experiment were shifted in time by about 24 h. This delay becomes more evident by comparing the CT and OT (see Fig. 11 c and d) which reached ~ 100 mmol L^−1^ at 96 h in the 1.5 L cultivation and at 120 h in the 10 L.

A comparison of the calculated tip-speed of the two fermentations over the course of the cultivation indicated that at 10 L scale the tip-sped was always lower than in the 1.5 L reactor (see Fig. 11e). The overlay of the calculated EDCF values (energy dissipation circulation function) of both processes indicated that the “fragmentation energy” the pellets experienced at 10 L scale was approximately > 10 times lower than at 1.5 L scale. The differences in the EDCF values were more pronounced at higher stirrer speeds since EDCF increases exponentially with the tip-speed. To simplify the calculation the following assumptions and simplifications have been applied: turbulent flow, the ungassed power number of the Rushton turbine was used and a density of water assumed for the culture broth. The power number (P) and the flow number (F) were taken from [27, 28].

## Discussion

Illudin M titers were previously improved by developing an optimized fed-batch process in shake flasks. It was concluded that the feeding of basic potassium-acetate as biosynthetic precursor influenced cell growth by a combination of acetate toxicity and impact of high pH, leading to an optimal ratio of active biomass to substrates at the production phase and a prolonged production time frame. It was assumed that by providing the biosynthetic precursor for isopentenyl pyrophosphate (IPP) biosynthesis directly to the mevalonate pathway, the carbon flux was channeled mostly into product and not biomass formation which, in combination with a feed of glucose for energy maintenance improved illudin M titers [8]. The data from the online monitoring of respiration activity from the batch and fed-batch shake flask cultures were consistent with these previous hypotheses since after feeding acetate the culture had a prolonged and higher respiratory activity compared to the batch process (see Figs. 1 and 2). To achieve similar process profiles in stirred tanks, we proposed biomass and morphology control as key factors for modulating process performance and to achieve titers comparable to the shake flask process. We found that pH per se influenced biomass formation in stirred tank cultivations since results from pH-controlled cultivations indicated that pH 4.5 promoted biomass growth while pH > 6 reduced biomass and influenced morphology (more distinct pellets). This was expected since many higher fungi grow better at low pH (< 5) and it is known for fungal submerged cultivations that pH strongly influences cell growth affecting cell wall function, enzymatic reactions and nutrients uptake [29]. Culture pH can also influence morphology in liquid cultures and it has been reported that higher pH promotes pellet formation which has the advantage of reduced broth viscosity compared to filamentous growth in the culture [10]. This effect was also observed with *O. nidiformis* (see Fig. 3)*.* Our results indicated that pH was a key parameter to control cell growth and morphology in the cultures and therefore a pH-control strategy was developed, considering pH 6.2 as lower boundary to avoid severe growth inhibition. Since pH shifts in the stirred tanks are realized by addition of acid or base their effect can be investigated independently of the acetate feed. This allowed—in contrast to the shake flask experiments—investigation of pH-shift time and pH-shift value as growth and morphology modulators. For this investigation the process was divided into three phases: growth phase (pH 4.5), biomass and morphology-control phase (pH 6.5) and production phase (pH 6.5) with acetate feeding. This scheme enabled the cultures to reach a suitable stage in which the feed of acetate did not inhibit the producer but strongly improved product titers. A comparable strategy was successfully applied to improve the production of itaconic acid in submerged cultivations of the ascomycete *Aspergillus terreus* since pH influenced product formation, cell growth and morphology. Similar as in our setup, low pH seemed to be crucial during the growth phase of *A. terreus*, and a timed shift of the pH in the production phase towards a higher value was key for improved itaconic acid production [30]. Higher culture pH was crucial to increase itaconic acid titers since the abundance of undissociated itaconic acid shifted to a minimal concentration and thereby avoiding growth inhibition by diffusion of itaconic acid into the cell and subsequent dissociation and accumulation in the neutral cytosol [31]. A strategy which we used to reduce the intracellular accumulation of dissociated acetate before it could be channeled into IPP biosynthesis.

We found that pH-shift time and pH-shift value influenced illudin M titers (see Figs. 5 and 6). Therefore, by screening different pH-shift times and pH-shift values in stirred tank bioreactors (DASGIP 1.5 L), we determined the optimal range of these parameters that enabled to establish a fully controlled process setup in which biomass and morphology where modulated to achieve illudin M titers comparable to the shake flask process (RAMOS). However, acetate consumption in stirred tank cultivations was more than doubled compared to the shake flasks. These changes were caused by the inherent differences of the two cultivation systems, which led to a higher biomass growth, probably caused by a different type of mixing of the culture in the case of the stirred tank. Our results showed that by adapting the cultivation and feeding strategies we could overcome the differences between cultivation systems and we were able to achieve similar process profiles by the time of the precursor feeding. This was reflected in similar OTR and CTR values until 96 h (Fig. 9a), which became clearer when comparing the overlay of the averaged OT and CT of several DASGIP and RAMOS experiments. After the batch phase, at the time of feeding (96 h) the CT and OT values were consistently ~ 100 mmol L^−1^ in both cultivation types, when high titers were reached.

It is probably no coincidence that 96 h is the empirically established optimal feeding time, which has been evaluated in shake flask without having the respiratory data available. The change in respiratory activity, which was monitored with the RAMOS, indicated a change in metabolic activity exactly at 96 h (see Fig. 1). We assume that this change in respiratory activity falls together with the initiation of the secondary metabolism and that the feeding at that point has the biggest impact on illudin M formation. We propose that CT and OT values ~ 100 mmol L^−1^ at 96 h should be used as indicator of the metabolic state of the culture, especially in bioreactors in which these changes in the CTR and OTR curves were less pronounced than in the RAMOS flasks. Deviations from those values could be used as an early predictor in our cultivations that the final titers would be below the highest observed ~ 1000 mg L^−1^.

The subsequent scale up experiment to 10 L scale showed similar kinetics for substrates consumption compared with the 1.5 L cultivation (Fig. 11b) but illudin M titers were significantly lower (see Fig. 11a). The CTR and OTR curves were slightly different since the values were lower and time-shifted in the 10 L cultivation (see Fig. 11a). This became obvious by comparing the CT and OT values which did not reach 100 mmol L^−1^ by the time of the first acetate feed but 24 h later (Fig. 11c, d) and this could be the reason for reduced illudin M production. The delayed development of the 10 L cultivation might have been caused by low stirring rates at the beginning of the cultivation leading to poor mixing. These results indicated that adaptation of process parameters is required to achieve a similar process profile as in the 1.5 L scale especially during the batch phase. It should be considered that the differences of the vessel at 10 L scale (larger vessel, baffles, ring sparger and different vessel to impeller diameter ratio) introduce changes in the bioreactor environment that might influence biomass growth and thereby productivity. Shear forces generated through agitation and aeration in stirred tanks are known to induce changes in the cell structure as well as changes in the morphology which influences cell growth [32]. Mechanical forces can cause pellet disruption or fragmentation into smaller units that can act as new growth centers, which accelerates biomass development [10]. It has been reported that the local hydromechanical stress caused by Rushton impellers can lead to a more efficient dispersion than the one observed in shake flasks under standard cultivation conditions [33, 34]. In stirred tanks a second form of hydromechanical stress is introduced by the aeration through a sparger. The combined effect of both forms of stress on product formation and morphology of the producer was studied in cultivations of *Aspergillus niger* applying different combinations of gassing rate and stirrer speed keeping the total volumetric power input constant [35]. It was found that power input from higher aeration contributed significantly to erosion of hyphae from the surface of the pellet and pellet disruption with two distinct effects: breakage of hyphae might have generated new centers for growth, and pellets were less compact and of irregular structure. As a consequence, cells benefited from better nutrient supply to the pellet center, contributing to more active cells and higher product formation. Correlating biomass formation and productivity from different experiments indicated that the highest quantity of biomass did not result in the highest product titers. The authors concluded that product formation requires an ideal quantity of active biomass which can be achieved by influencing morphology through modulation of hydromechanical stress [35], a finding that underlines our hypothesis of the optimal biomass to nutrient ratio at the onset of production.

In depth investigations of morphological changes were not in the scope of this work, yet we observed obvious differences between the appearances of the pellets from the shake flask and stirred tank process. Pellets from stirred tank cultivations seemed less compact and smaller than the pellets from shake flasks that appeared to be rounder, more compact and larger as result of the differences introduced by the two different cultivation systems (see pictures in Additional file 1: Figs. S5, S6). It is plausible that under the established cultivation conditions in the bioreactor both, the aeration an the agitation had an impact on the structure of the pellets causing changes in pellet size and in pellet density leading to looser and smaller pellets compared to the shake flask process and thereby increased biomass. As a consequence the wet biomass measured at harvest represented 6% of the total volume of the shake flask cultures and 10% in the case of the stirred tank. These increased quantities of biomass in the stirred tank contributed to the observed higher oxygen and acetate consumption which have been discussed above and were compensated by an adapted feeding scheme.

A commonly discussed concept for scaling up cultivations of filamentous organisms in stirred tanks is the energy dissipation circulation function (EDCF), which does not consider only the energy introduced by the tip speed of the impeller into the culture, but also the exposure time of the pellet to this energy. The applicability of this concept has been shown in several examples [13, 14] and the commonly observed phenomena that the shear stress is actually reduced in up-scaled cultivations can be explained with good correlations. Figure 11e, f show the comparison of the tip speed of the 1.5 L and 10 L fermentations, which was significantly lower in the latter. Comparison of the EDCF which was calculated for the two cultivations showed that the values estimated for the 10 L cultivation were by a factor of 10 lower than in the 1.5 L experiment indicating highly reduced hydromechanical stress in the larger cultivation volume. This might be a potential explanation for the time-shifted process kinetics in the 10 L cultivation since higher shear stress is commonly associated with more fragmentation of hyphae and generation of new growth centers [35–37]. This finding implies that shear stress might be of use to modify the growth behavior in the batch phase but will be of less importance and can be probably neglected in the production phase when it comes to larger cultivation volumes. Yet the strain will potentially be exposed to higher dissolved oxygen concentrations since the oxygen transfer efficiency increases with the volume and the oxygen demand of the strain might not be sufficient to bring the pO_2_ value down to 5%. Our results indicate that the batch phase of the process might be crucial to bring the culture into a condition where the provided precursors have the biggest impact on product formation. Effects introduced by the different cultivation system which became more apparent in the feeding/production phase when comparing off-gas data, could be counterbalanced by an adapted feeding strategy. It seems that during the production phase the strain is more tolerant to deviations from the “optimal” culture course derived from the shake flask process. Differences from this “optimal course” seem to have a higher impact when occurring in the batch phase which can be easily monitored observing the CT and OT values. We have introduced parameters that allow to control the growth and morphology to a certain extent and propose additional factors which might become important in further scale up. Our results indicated that it is difficult to transfer a process from shake flasks into stirred tanks considering only a single parameter. Yet knowledge which can be generated with shaken systems and a careful observation of the culture during the process, especially when changing conditions helps to understand the behavior of the strain and how these effects can be used to modulate growth towards an improved biomass to nutrient ratio.

For further scale up we propose a seed fermentation in the batch medium at low pH, with low aeration rates and DO control at 5–10% which results in high stirring rates which “homogenizes” the biomass. The ideal inoculum size must be investigated in further studies. In the production culture special emphasis should be given to the batch phase which seemed to be crucial to prepare the strain for a successful later production phase. We propose to put special emphasis on the CT and OT values, which should reach both about 100 mmol L^−1^ at 96 h of cultivation time. This is the proposed onset of secondary metabolism and growth should be carefully monitored (CT and OT) and modulated by applying a carefully timed pH shift towards higher values when a reduced growth rate is necessary. If an initial lag phase as in the 10 L cultivation is observed, a later pH shift combined with an increased stirring rate in the batch phase and/or a larger inoculum could lead to the optimal concentration of biomass at the time point of the first feeding. The strain seemed to be less influenced from physicochemical changes once the secondary metabolism is upregulated, but the better mass transfer in larger stirred tanks might lead to higher oxygen concentrations in the course of the production and this factor might be worth to be investigated beforehand when scaling up to larger cultivation volumes.

### Limitations and technical challenges of the cultivations in stirred tank bioreactors

We have realized several important points that strongly influenced the process performance in stirred tank reactors, which required considerable attention to achieve comparable results in all experiments. Discharge of biomass from inside the liquid into the surface/vessel head space, could occur due to air bubbles introduced by the sparger. The small bubbles can carry biomass to the gas–liquid interface and when bursting in proximity to the vessel wall or built in parts, biomass is spilled above the surface of the liquid. This can accumulate over time reducing the quantity of biomass that actively grows within the liquid phase, which is of special importance at the beginning of the cultivation when cell growth is slow due to a lag phase following inoculation. In our process transfer experiments the constant addition of small quantities of antifoam from the start of the cultivation reduced this effect and enabled reliable results.

An additional important detrimental effect can happen when larger biomass clumps form in the culture, and get stuck between built-in parts of the vessel. Once stuck, the clumps usually lead to further accumulation of biomass at that point with extensive growth at later stages. This is particularly critical when the DO sensor is in close proximity or surrounded by such a “growth center” since the measured values influence the regulation of dissolved oxygen. Therefore, false values for the dissolved oxygen might result in high stirring rates or increased aeration cascades, leading to disturbed process conditions and making data evaluation difficult. Special care has to be taken when assembling the built-in accessories of the vessel to avoid narrow spaces between accessories or the vessel wall. The occurrence and severity of these effects is dependent on many factors and unpredictable. Observation of the culture at the gas/liquid interface through a sight glass is helpful, and may reveal these issues for proper intervention. We assume that these effects have a greater importance or effect in small scale experiments, as spatial limitations are less prone in larger vessels and the ratio of the area above the liquid surface (where biomass can accumulate) to culture volume, is less in larger bioreactors.

## Conclusion

We transferred the process from shaken into stirred tank bioreactors using the recently developed medium, adapting the feeding of acetate as a biosynthetic precursor and by establishing a growth control scheme using timed pH-changes. The three step control strategy was considered necessary since the conditions in the bioreactor influenced the morphology of *O. nidiformis* and the growth. In 1.5 L stirred tank cultivations titers around > 900 mg L^−1^ were achieved, comparable to the titers obtained in the optimized shake flask process. Finally, by transferring the findings from1.5 L scale to 10 L, titers > 500 mg L^−1^ were achieved. Since several factors like shear rate and oxygen entry, which are vessel specific, contribute to the growth of the organism, an adapted growth control strategy might be necessary in a different vessel and potential reasons have been discussed and solution strategies proposed. This can theoretically result in higher or lower pH values and/or earlier or later shift times than in our reported case. As a benchmark, an early 10 L cultivation in a stirred tank bioreactor without improved medium and under conditions typically used for cultivation of filamentous fungi, reached a concentration of 44 mg L^−1^(see supplementary information, additional file 1: Fig. S7), which is by a factor of 12 lower than the result achieved with a single educated experiment. The highest illudin M titer previously reported in stirred tank bioreactors was ~ 88 mg L^−1^ after 164 h, however there was no disclosure of a detailed process development to allow reproduction of that process [38]. Our study has not just surpassed that titer but provides a detailed methodology to produce high titers of illudin M in stirred tank bioreactors, and the presented approach might be transferable to similar processes for the production of other fungal compounds. Our processes at 1.5 L and 10 L provide a solid starting point for further optimization and are sufficient to enable early phase supply of new development campaigns for illudin M derivatives.

## Methods

Strain maintenance and preparation of liquid cultures in conventional shake flasks have been previously described and named as method SP4 [8]. However, a simplified description is presented in this work for clarity. Analytical methods for quantification of product and substrates were identical to those reported in the above mentioned publication. For all experiments in stirred tank bioreactors, control shake flask cultivations (optimized fed-batch process) were performed in parallel using the same inoculum to ensure that outcomes from the fermentations were not related to problems with the seed culture or fitness of the strain but related to the evaluated process setup.

### Strain, media and supplements

All cultivations were performed using the strain *Omphalotus nidiformis* DSM23613 maintained on agar plates of YM 6.3 medium at 23 °C in darkness. The culture medium for submerged cultivations was G13.5/C7 and the modified Czapek-Dox solution was added to the medium prior sterilization. The composition of the media are listed in Table 3. The stock solutions used for feeding are listed in (Table 4).

**Table 3:** Culture media used during the study

Culture medium	Components and concentrations (g L^−1^)
YM 6.3	Malt extract (10), Glucose (4), Yeast extract (4), agar (20), pH 6.3
G13.5/C7	Glucose monohydrate (13.5), Corn Steep Solids (7), Czapek-Dox Broth^a^ (35 mL)
Czapek-Dox broth^a^	NaNO_3_ (1), KH_2_PO_4_ (1), MgSO_4_.7H_2_O (0.5), KCl (0.5), FeSO_4_.7H_2_O (0.01)

**Table 4:** Stock solution for feeding

Stock solution(g L^−1^)	Components and concentrations (g L^−1^)
Glucose (360)	Glucose monohydrate (396)
Acetate (600)	Potassium acetate (980)

### Seed preparation

The strain *O. nidiformis*, was cultivated in agar plates of the medium YM 6.3 and incubated at 23 °C for 3 weeks in darkness. Liquid seed cultures were inoculated with ten agar plugs from the outer edge of the mycelium and homogenized at 8000 min^−1^ for 1 min. Cultures were incubated for 5 days at 23 °C and 160 min^−1^ shaking speed (orbit diameter 50 mm).

### Inoculation of main cultures

The seed culture was homogenized at 8000 min^−1^ for 1 min and transferred into weighed sterile 50 mL Falcon™ tubes. Tubes were centrifuged at 4300×*g* for 15 min using an Eppendorf® Centrifuge 5804R. After centrifugation, the liquid phase was discarded keeping only the complete biomass (top layer and pellet). The weight of the biomass was determined, and fresh culture medium was added to the tubes to reach a final concentration of 200 g L^−1^ biomass. The biomass suspension was well mixed by homogenizing for 10 s at 8000 min^−1^. The homogenized suspension was pooled in a sterile glass beaker and continuously stirred at 500 min^−1^ with a magnetic stirrer during inoculation. Main cultures were inoculated at 1 g L^−1^ biomass.

### Monitored shake flasks cultivations

Cultivations were performed with the Respiration Activity Monitoring System -RAMOS® (HiTec Zang GmbH, Germany) in order to monitor the course of oxygen transfer rate (OTR) and carbon dioxide transfer rate (CTR). Production cultures were prepared with 200 mL of medium in 500 mL RAMOS® flasks. Cultures were incubated in the integrated orbital shaker Climo-Shaker ISF1-X (Kuhner AG, Switzerland) at 23 °C and 160 min^−1^ shaking speed (50 mm orbit diameter). Aeration rate was set to 4.7 mL min^−1^ using compressed dry air. Potassium acetate (CH_3_CO_2_K) was fed at 96 h to reach a final concentration of 8 g L^−1^ acetate in the cultures, and 6 g L^−1^ of glucose were fed at 120 h.

### Cultivations in 2 L stirred tank bioreactors

Cultivations were performed in small scale stirred tanks DASGIP® (Eppendorf, Germany), consisting of 2 L glass vessels equipped with optical DO sensors (Hamilton, Germany), pH electrodes (Hamilton, Germany) and temperature sensor. Gas was supplied through an L-sparger centrally placed (clearance of 20 mm between the bottom of the vessel and sparger). The Off-gas analysis were performed with zirconium dioxide sensors (ZrO_2_) for O_2_ measurement, and infrared (IR) sensors (BlueSens, Germany) for CO_2_ measurement. Process was controlled by DASware® Control 5.6.2 (Eppendorf, Germany). Equipment dimensions are described in Table 5.

**Table 5:** Dimensions of equipment used for 1.5 L cultivations (DASGIP)

Dimension	Value
Vessel inner diameter	10 cm
Vessel length	30 cm
Type of impeller	Rushton
Number of impellers	3
Distance between impellers*	4 cm
Impeller diameter	5.5 cm
Impeller/vessel diameter	0.55
Max. liquid volume	1.5 L

*Lowest impeller was placed 4.5 cm from the bottom of the vessel

Cultivation volume was 1500 mL including inoculum. The pH was maintained at set point values by addition of sulfuric acid (H_2_SO_4_ 10% stock solution) and potassium hydroxide (KOH 10% stock solution). Dissolved oxygen (DO) was controlled by increasing the stirrer speed (200–1000 min^−1^). Foaming was controlled by the addition of antifoam SB253 Struktol® (Schill + Seilacher, Germany) at a rate of 20 µL h^−1^ through an automated syringe pump. Initial pH was set to 4.5, temperature was controlled at 23 °C, DO 5% and gassing rate 27 L h^−1^ (0.3 vvm).

### Cultivation in 15 L bioreactors

Cultivations were performed in a 15 L stainless steel vessel with 4 baffles (xCUBIO in-situ, bbi biotech, Germany). The bioreactor was equipped with sensors for temperature, level and foam as well as sensors for DO and pH (Hamilton, Germany). Gassing was performed through a ring sparger with 15 mm clearance from the bottom of the vessel. The off-gas analysis were performed with zirconium dioxide (ZrO_2_) sensors for O_2_ and infrared (IR) sensors for CO_2_ (BlueSens, Germany). Process was controlled by xCUBIO control software. The dimensions of the vessel are listed in Table 6.

**Table 6:** Dimensions of equipment used for 10 L cultivations

Dimension	Value
Vessel inner diameter	18.5 cm
Vessel length	56 cm
Type of impeller	Rushton
Number of impellers	3
Distance between impellers*	10.5 cm
Impeller diameter	7.5 cm
Impeller/vessel diameter	0.40
Max. liquid volume	10 L

*Lowest impeller was placed 6 cm from the bottom of the vessel

DO and pH were controlled in the same way as in the 1.5 L cultivations in DASGIP®. The antifoam SB253 was fed at a rate of 100 µL h^−1^ to prevent foaming. Initial pH was set to 4.5, temperature was controlled at 23 °C, DO 5% and gassing rate 180 L h^−1^ (0.3 vvm).

### Final feeding scheme in stirred tank cultivations

Acetate was fed three times during the cultivation time at 96 h (8 g L^−1^), 120 h (4 g L^−1^) and 144 h (4 g L^−1^). Glucose was fed one time at 120 h (6 g L^−1^).

### Calculation of the energy dissipation circulation function (EDCF)

The equations used to calculate the EDCF were previously reported [37] and are listed in Table 7.

**Table 7:** Equations used for determination of the EDCF and description of terms with respective units

Equation	Nomenclature
$k=\left(\frac{\pi }{4}\right)\left(\frac{W}{D}\right)$	k: impeller geometrical factor W: impeller blade height (m) D: impeller diameter (m) P: power input (W) P_0_: ungassed power number of the impeller ρ: broth density (kg m^−3^) 1/t_c_: circulation frequency (s^−1^) Fl: impeller flow number N: stirring speed (s^−1^) V_L_: working volume (m^3^)
$$P= {P}_{o }\rho {N}^{3}{D}^{5}$$	
$$\frac{1}{{t}_{c}}=\frac{(FlN{D}^{3})}{{v}_{l}}$$	
$$EDCF= \left(\frac{P}{k{D}_{3}}\right) \left(\frac{1}{{t}_{c}}\right)$$	

## Supplementary Information

**Additional file 1: Fig. S1.** Relative abundance of undissociated acetate at different pH values. **Fig. S2.** Process kinetics from 1.5 L cultivations in stirred tanks where pH was shifted from pH 4.5 to pH 6.5 at four different cultivation times. **Fig. S3.** Process kinetics from 1.5 L cultivations in stirred tanks where pH was shifted from pH 4.5 to two different higher pH values at three different cultivation times. **Fig.S4.** Process kinetics from 1.5 L cultivations in stirred tanks for the screening of different pH values and pH shift times. **Fig.S5.** Appearance of pellets in samples of submerged cultivations of *Omphalutus nidiformis*. **Fig.S6.** Microscopic appearance of pellets from submerged cultivations of *Omphalutus nidiformis.* **Fig.S7.** Process kinetics from a non-optimized 10 L cultivation in a stirred tank for the production of illudin M.

## Data Availability

All data generated or analyzed during this study are included in this published article and its supplementary information files.

## Abbreviations

RAMOS: Respiration activity monitoring system
CTR: Carbon dioxide transfer rate
OTR: Oxygen transfer rate
CT: Integrated carbon dioxide transfer rate
OT: Integrated oxygen transfer rate
DO: Dissolved oxygen
vvm: Gas volume flow per unit of liquid volume per minute
EDCF: Energy dissipation circulation function
