# Supplementary material for: Optimization of the production process for the anticancer lead compound illudin M: process development in stirred tank bioreactors
Source: Microb Cell Fact. 2022 Jul 18;21:145. doi: 10.1186/s12934-022-01870-w (PMC9290264; doi:10.1186/s12934-022-01870-w)
Supplement: Supplementary file 1 — Additional file 1: Fig. S1. Relative abundance of undissociated acetate at different pH values. Fig. S2. Process kinetics from 1.5 L cultivations in stirred tanks where pH was shifted from pH 4.5 to pH 6.5 at four different cultivation times. Fig. S3. Process kinetics from 1.5 L cultivations in stirred tanks where pH was shifted from pH 4.5 to two different higher pH values at three different cultivation times. Fig.S4. Process kinetics from 1.5 L cultivations in stirred tanks for the screening of different pH values and pH shift times. Fig.S5. Appearance of pellets in samples of submerged cultivations of Omphalutus nidiformis. Fig.S6. Microscopic appearance of pellets from submerged cultivations of Omphalutus nidiformis. Fig.S7. Process kinetics from a non-optimized 10 L cultivation in a stirred tank for the production of illudin M. [file 12934_2022_1870_MOESM1_ESM.pdf]

Additional file 1

Optimization of the production process for the anticancer lead compound illudin M: Process development in stirred tank bioreactors

Lillibeth Chaverra-Muñoz[1, 2], Stephan Hüttel*[1, 2]

1. Department of Microbial Drugs, Helmholtz Centre for Infection Research, Brunswick, Germany

2. German Centre for Infection Research (DZIF), Partner Site Hannover-Braunschweig, Brunswick, Germany.

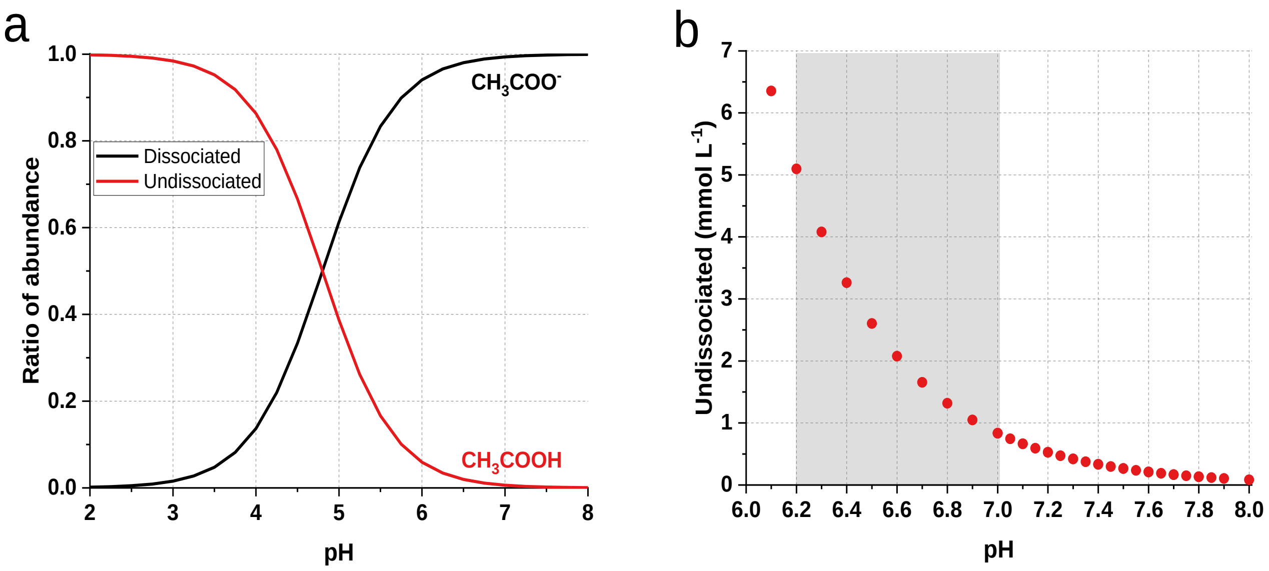

Fig. S 1 Relative abundance of undissociated acetate at different pH. a Calculated ratio of dissociated and undissociated acetate in aqueous solution from pH 2 to pH 8. **b** Calculated concentration of undissociated acetate based on a feeding concentration of 8 g L^{-1}. The gray area highlights the concentrations tested in our experiments.

**Due to the large amount of data and the size of the graphs, the set of figures for the following experiments is divided into several pages. Figures of the same experiment are labelled with the same legend (Fig.Sx) but the reference to specific graphs is annotated for each subset (a, b, c etc.).

Set of experiments for pH shift time (24 h, 48 h, 72 h and 96 h)

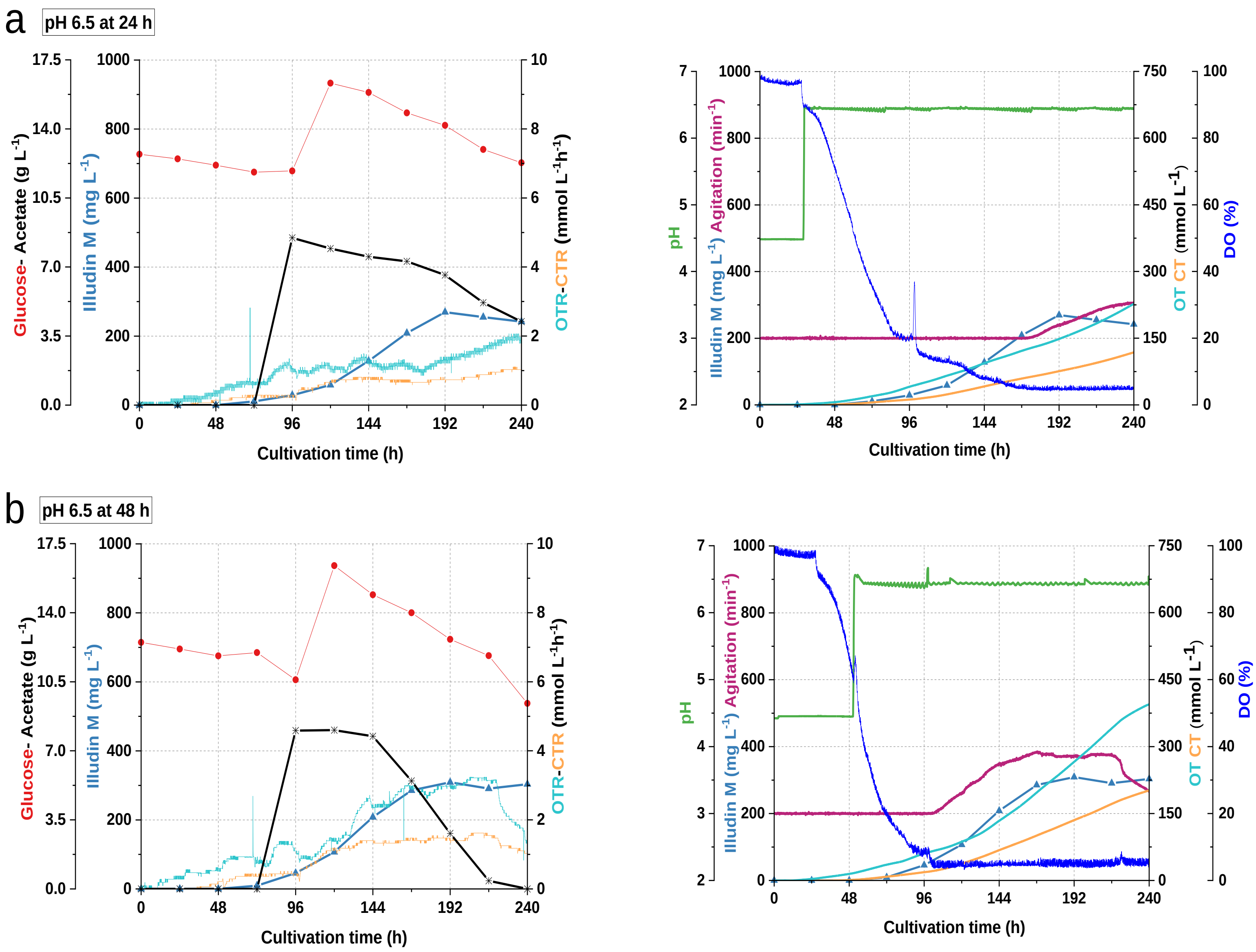

Fig. S 2 Process kinetics from 1.5 L cultivations in stirred tanks where pH was shifted from pH 4.5 to pH 6.5 at four different cultivation times. All cultures were prepared with *O. nidiformis* cultivated in G13.5/C7 medium at 23°C, 0.3 vvm (27 sL h^{-1}) aeration and dissolved oxygen (DO) was maintained at 5% by increasing the stirring speed. Acetate (8 g L^{-1}) was fed at 96 h and glucose (6 g L^{-1}) at 120 h. All curves illustrating the course of the different process parameters are colored according to the colors of the axis labels. **a** shift time 24 h **b** shift time 48 h. Illudin M titers were derived from cell free culture supernatant.

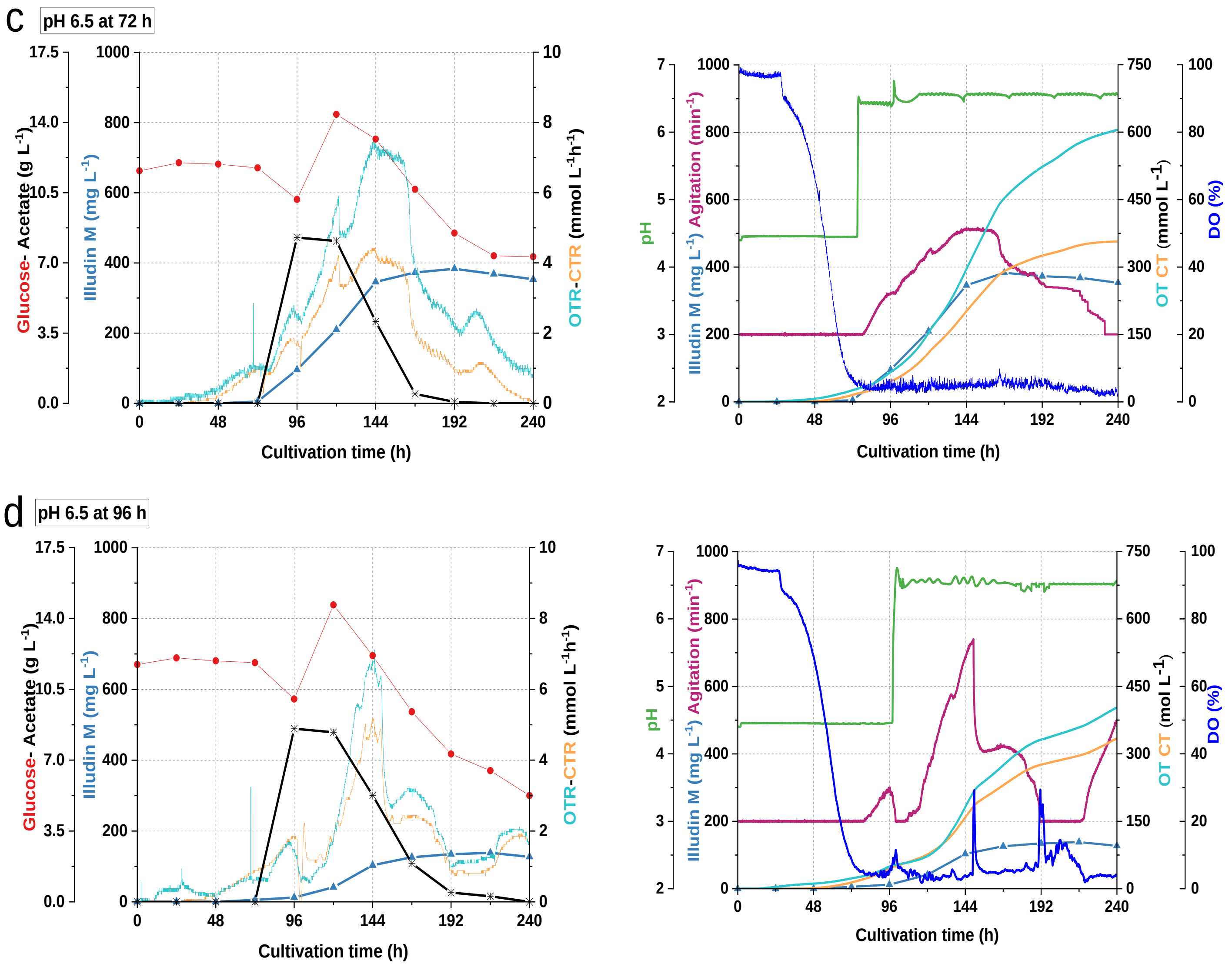

Fig. S 2 Process kinetics from 1.5 L cultivations in stirred tanks where pH was shifted from pH 4.5 to pH 6.5 at four different cultivation times. All cultures were prepared with *O. nidiformis* cultivated in G13.5/C7 medium at 23°C, 0.3 vvm (27 sL h^{-1}) aeration and dissolved oxygen (DO) was maintained at 5% by increasing the stirring speed. Acetate (8 g L^{-1}) was fed at 96 h and glucose (6 g L^{-1}) at 120 h. All curves illustrating the course of the different process parameters are colored according to the colors of the axis labels. **c** shift time 72 h (this culture achieved the highest illudin M titer) and **d** shift time 96 h. Illudin M titers were derived from cell free culture supernatant.

Set of experiments for pH shift time (63 h, 72 h and 81 h) and pH value (6.5 or 6.8)

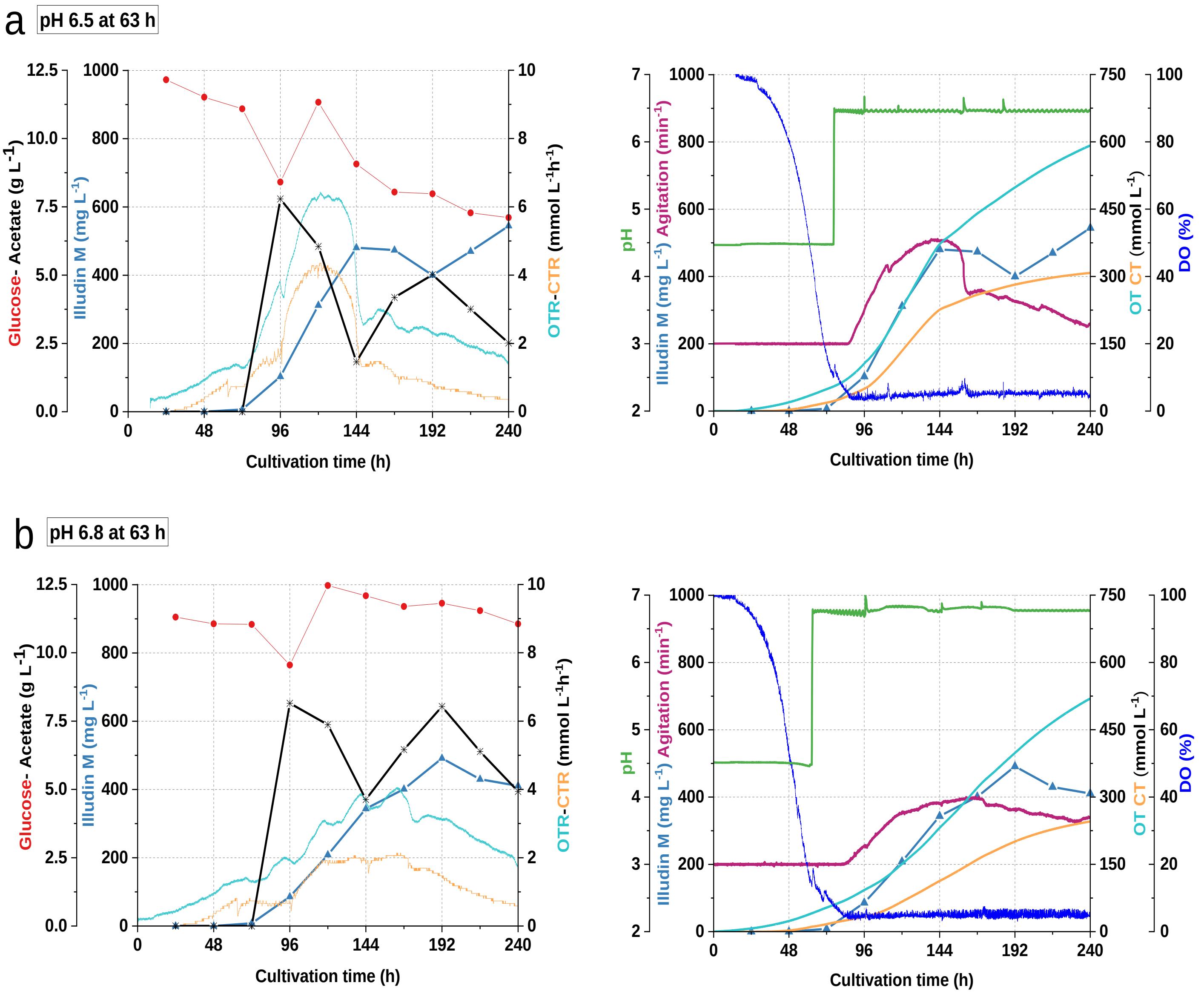

Fig. S 3 Process kinetics from 1.5 L cultivations in stirred tanks where pH was shifted from pH 4.5 to two different higher pH values at three different cultivation times. All cultures were prepared with *O. nidiformis* cultivated in G13.5/C7 medium at 23°C, 0.3 vvm (27 sL h^{-1}) aeration and dissolved oxygen (DO) was maintained at 5% by increasing the stirring speed. Acetate was fed at 96 h (8 g L^{-1}), 168 h (4 g L^{-1}) and 192 h (4 g L^{-1}). A feed of glucose was performed at 120 h (6 g L^{-1}). All curves illustrating the course of the different process parameters are colored according to the colors of the axis labels. **a** pH shifted to 6.5 at 63 h. **b** pH shifted to 6.8 at 63 h. Illudin M titers were derived from cell free culture supernatant.

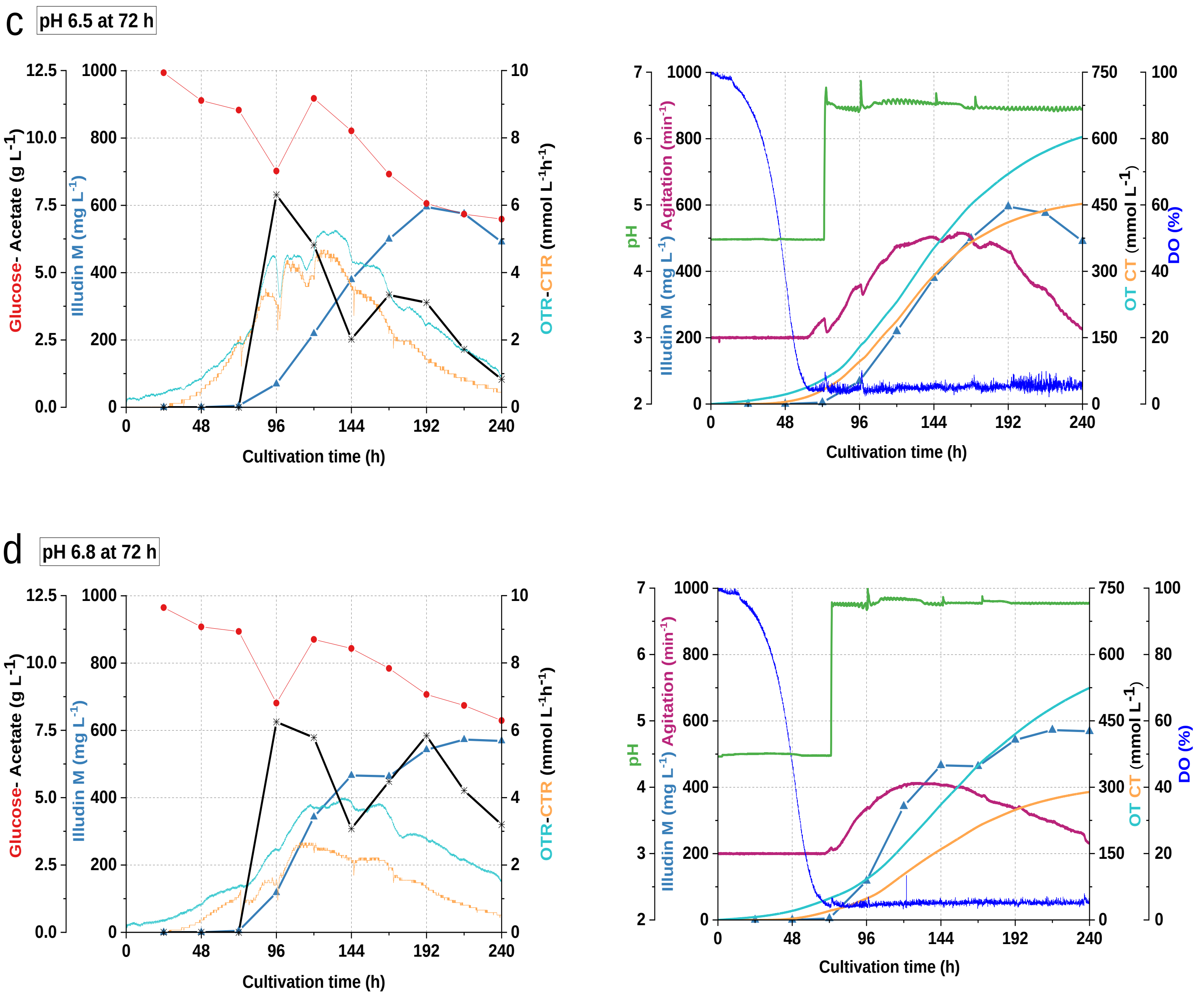

Fig. S 3 Process kinetics from 1.5 L cultivations in stirred tanks where pH was shifted from pH 4.5 to two different higher pH values at three different cultivation times. All cultures were prepared with *O. nidiformis* cultivated in G13.5/C7 medium at 23°C, 0.3 vvm (27 sL h^{-1}) aeration and dissolved oxygen (DO) was maintained at 5% by increasing the stirring speed. Acetate was fed at 96 h (8 g L^{-1}), 168 h (4 g L^{-1}) and 192 h (4 g L^{-1}). A feed of glucose was performed at 120 h (6 g L^{-1}). All curves illustrating the course of the different process parameters are colored according to the colors of the axis labels. **c** pH shifted to 6.5 at 72 h. **d** pH shifted to 6.8 at 72 h. Illudin M titers were derived from cell free culture supernatant.

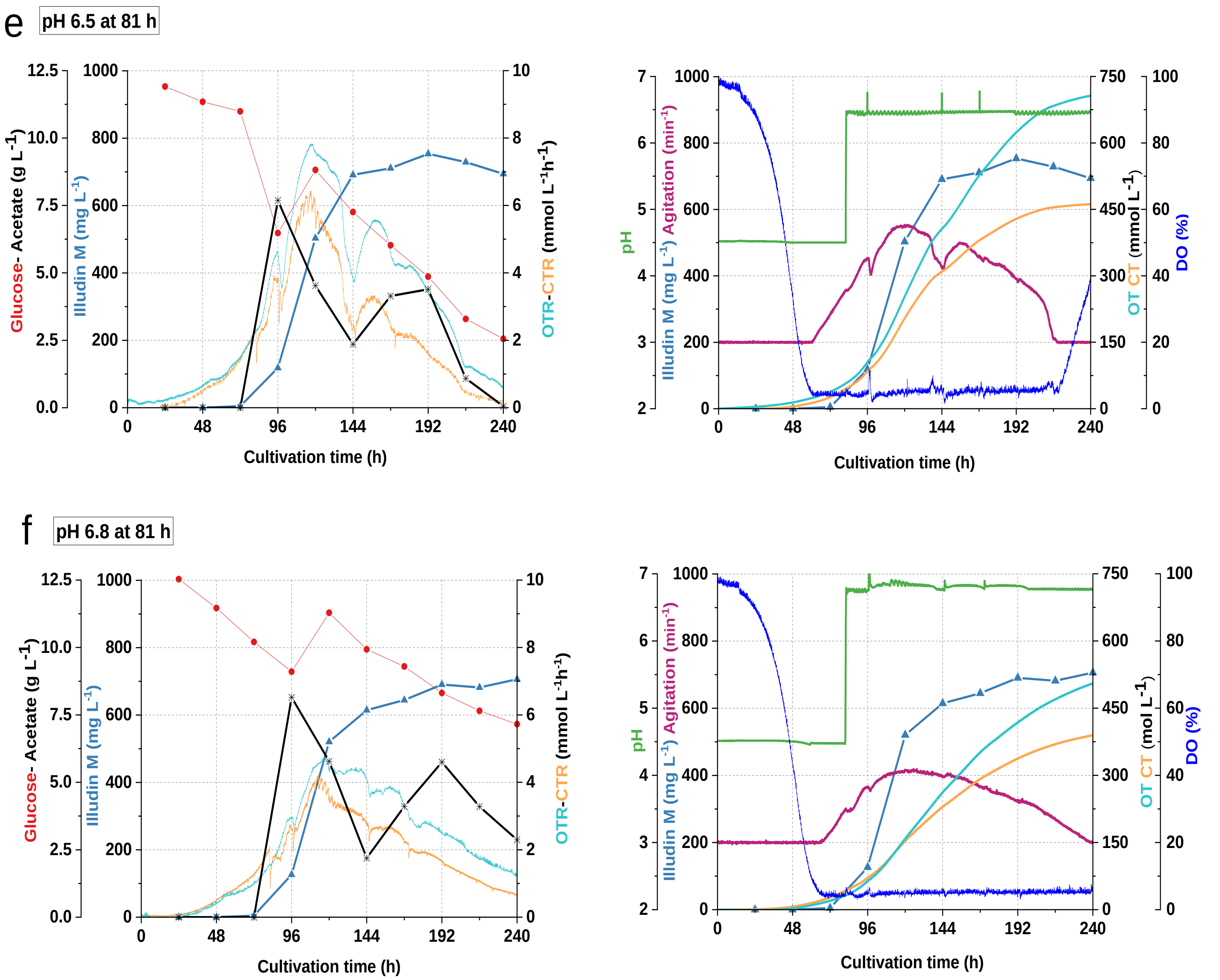

Fig. S 3 Process kinetics from 1.5 L cultivations in stirred tanks where pH was shifted from pH 4.5 to different higher pH values at three different cultivation times. All cultures were prepared with *O. nidiformis* cultivated in G13.5/C7 medium at 23°C, 0.3 vvm (27 sL h^{-1}) aeration and dissolved oxygen (DO) was maintained at 5% by increasing the stirring speed. Acetate was fed at 96 h (8 g L^{-1}), 168 h (4 g L^{-1}) and 192 h (4 g L^{-1}). A feed of glucose was performed at 120 h (6 g L^{-1}). All curves illustrating the course of the different process parameters are colored according to the colors of the axis labels. **e** pH shifted to 6.5 at 81 h (this culture achieved the highest illudin M titer). **f** pH shifted to 6.8 at 81 h. Illudin M titers were derived from cell free culture supernatant.

Set of screening experiments for optimal pH shift time and pH value

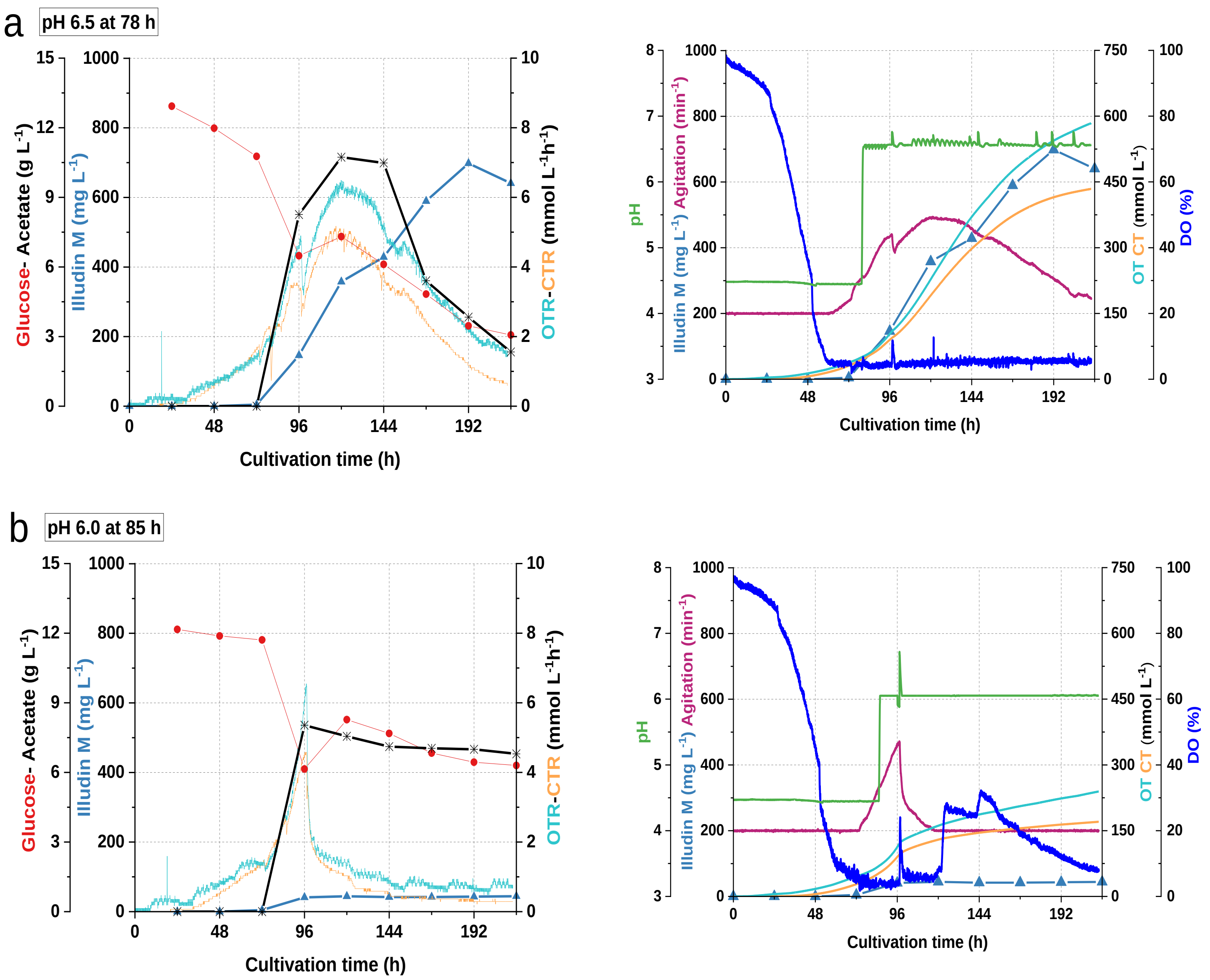

Fig. S 4 Process kinetics from 1.5 L cultivations in stirred tanks for the screening of different pH values and pH shift times. All cultures were prepared with *O. nidiformis* cultivated in G13.5/C7 medium at 23°C, 0.3 vvm (27 sL h^{-1}) aeration and dissolved oxygen (DO) was maintained at 5% by increasing the stirring speed. Acetate was fed at 96 h (8 g L^{-1}), 120 h (4 g L^{-1}) and 144 h (4 g L^{-1}). A feed of glucose was performed at 120 h (6 g L^{-1}). All curves illustrating the course of the different process parameters are colored according to the colors of the axis labels. **a** pH shifted to 6.5 at 78 h. **b** pH shifted to 6.0 at 85 h. Illudin M titers were derived from cell free culture supernatant.

c pH 6.25 at 85 h

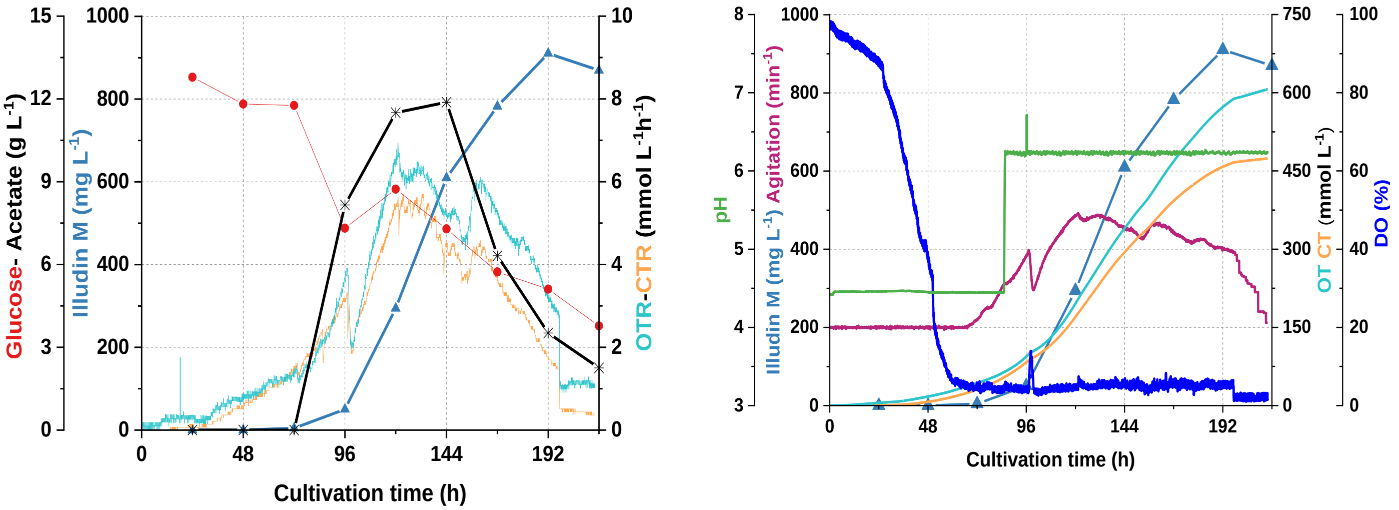

d pH 6.5 at 85 h

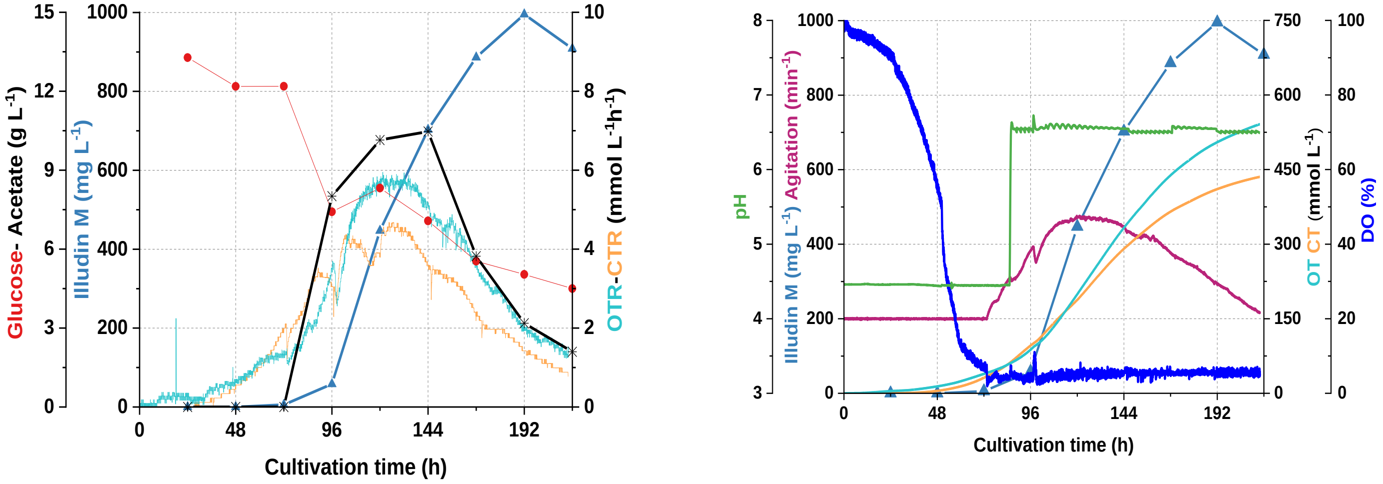

Fig. S 4 Process kinetics from 1.5 L cultivations in stirred tanks for the screening of different pH values and pH shift times. All cultures were prepared with *O. nidiformis* cultivated in G13.5/C7 medium at 23°C, 0.3 vvm (27 sL h^{-1}) aeration and dissolved oxygen (DO) was maintained at 5% by increasing the stirring speed. Acetate was fed at 96 h (8 g L^{-1}), 120 h (4 g L^{-1}) and 144 h (4 g L^{-1}). A feed of glucose was performed at 120 h (6 g L^{-1}). All curves illustrating the course of the different process parameters are colored according to the colors of the axis labels. **c** pH shifted to 6.25 at 85 h. **d** pH shifted to 6.5 at 85 h (this cultured reached the highest illudin M titer). Illudin M titers were derived from cell free culture supernatant.

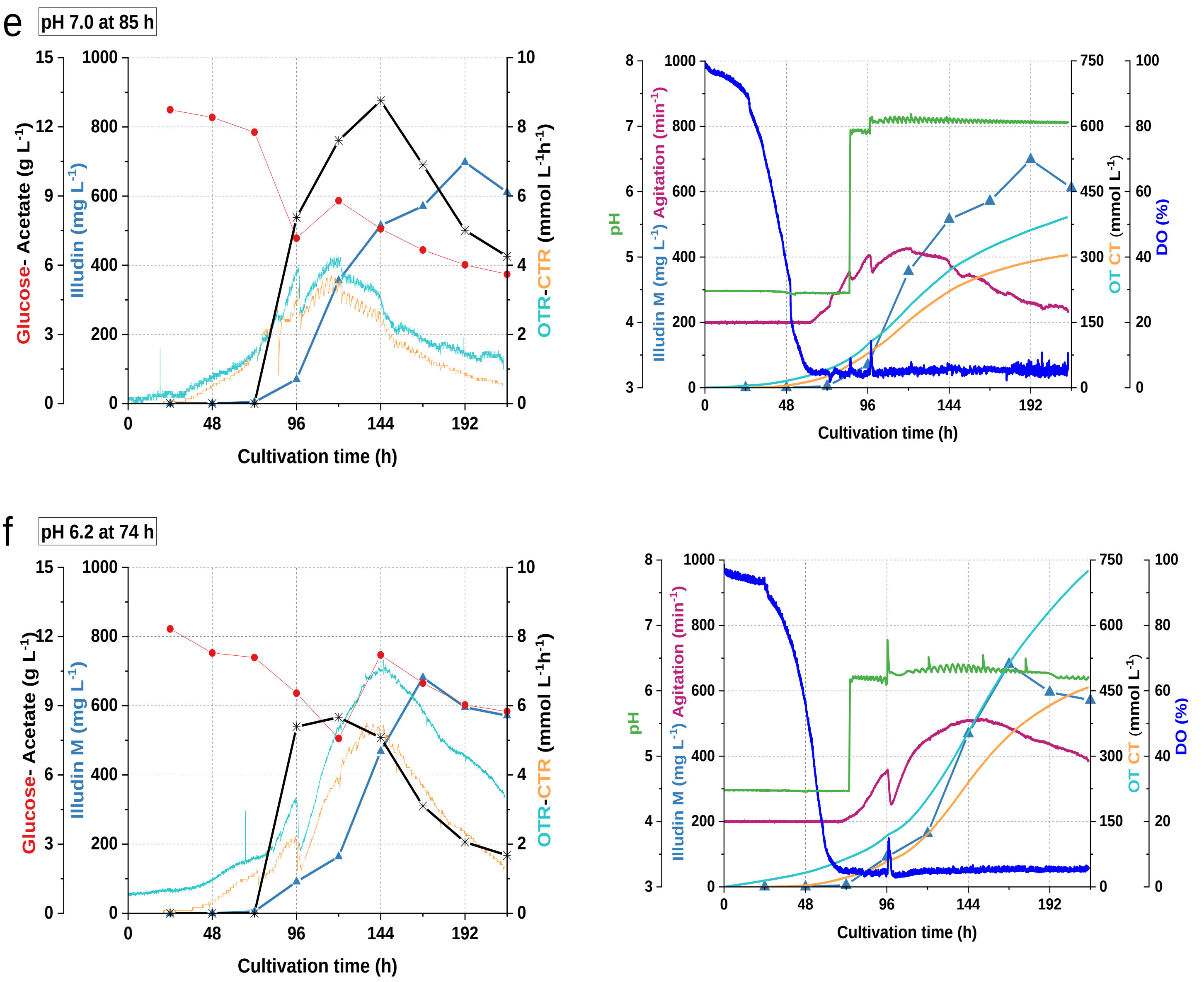

Fig. S 4 Process kinetics from 1.5 L cultivations in stirred tanks for the screening of different pH values and pH shift times. All cultures were prepared with *O. nidiformis* cultivated in G13.5/C7 medium at 23°C, 0.3 vvm (27 sL h^{-1}) aeration and dissolved oxygen (DO) was maintained at 5% by increasing the stirring speed. Acetate was fed at 96 h (8 g L^{-1}), 120 h (4 g L^{-1}) and 144 h (4 g L^{-1}). A feed of glucose was performed at 120 h (6 g L^{-1}). All curves illustrating the course of the different process parameters are colored according to the colors of the axis labels. **e** pH shifted to 7.0 at 85 h. **f** pH shifted to 6.2 at 74 h. Illudin M titers were derived from cell free culture supernatant.

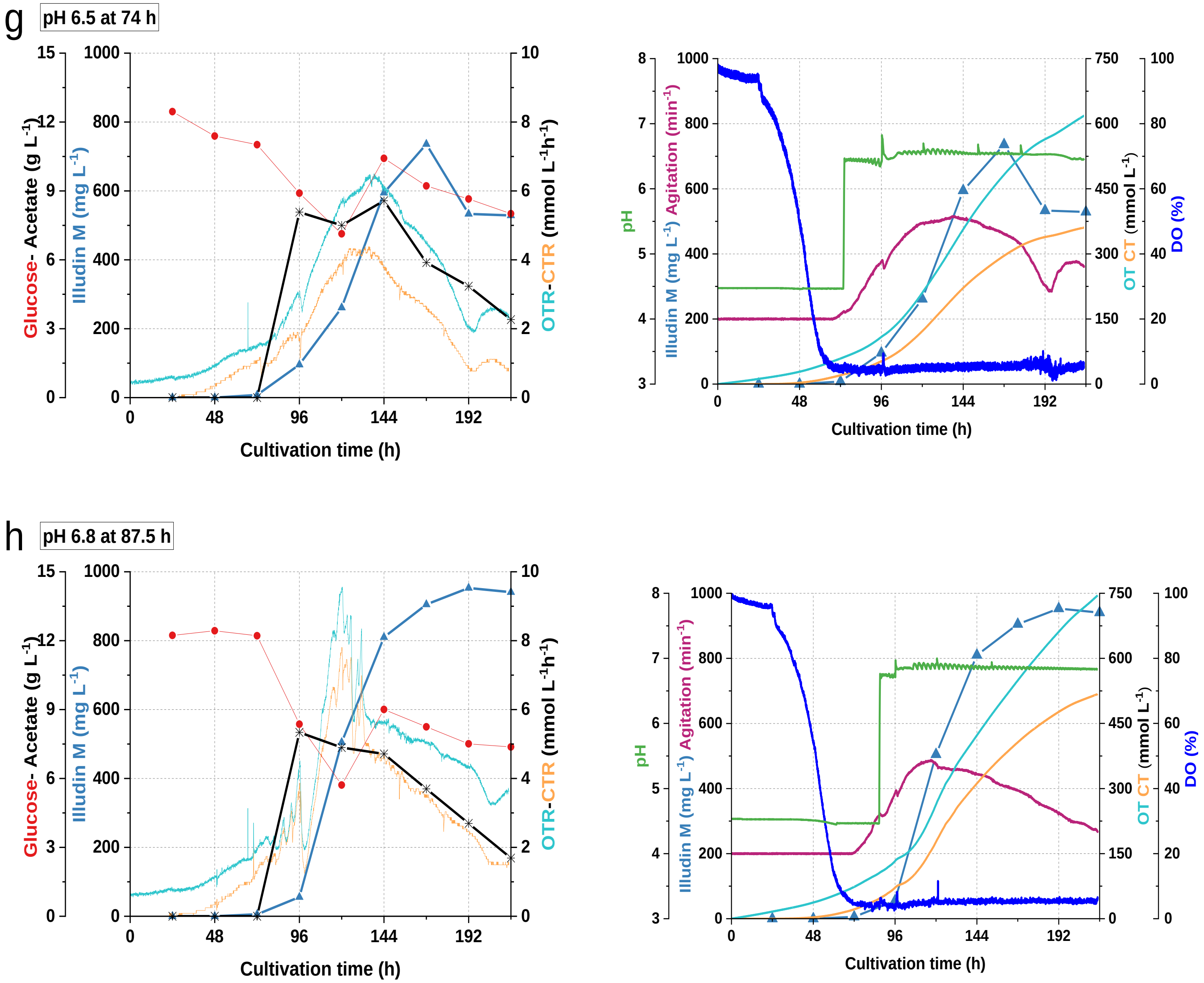

Fig. S 4 Process kinetics from 1.5 L cultivations in stirred tanks for the screening of different pH values and pH shift times. All cultures were prepared with *O. nidiformis* cultivated in G13.5/C7 medium at 23°C, 0.3 vvm (27 sL h^{-1}) aeration and dissolved oxygen (DO) was maintained at 5% by increasing the stirring speed. Acetate was fed at 96 h (8 g L^{-1}), 120 h (4 g L^{-1}) and 144 h (4 g L^{-1}). A feed of glucose was performed at 120 h (6 g L^{-1}). All curves illustrating the course of the different process parameters are colored according to the colors of the axis labels. **g** pH shifted to 6.5 at 74 h. **h** pH shifted to 6.8 at 87.5 h. Illudin M titers were derived from cell free culture supernatant.

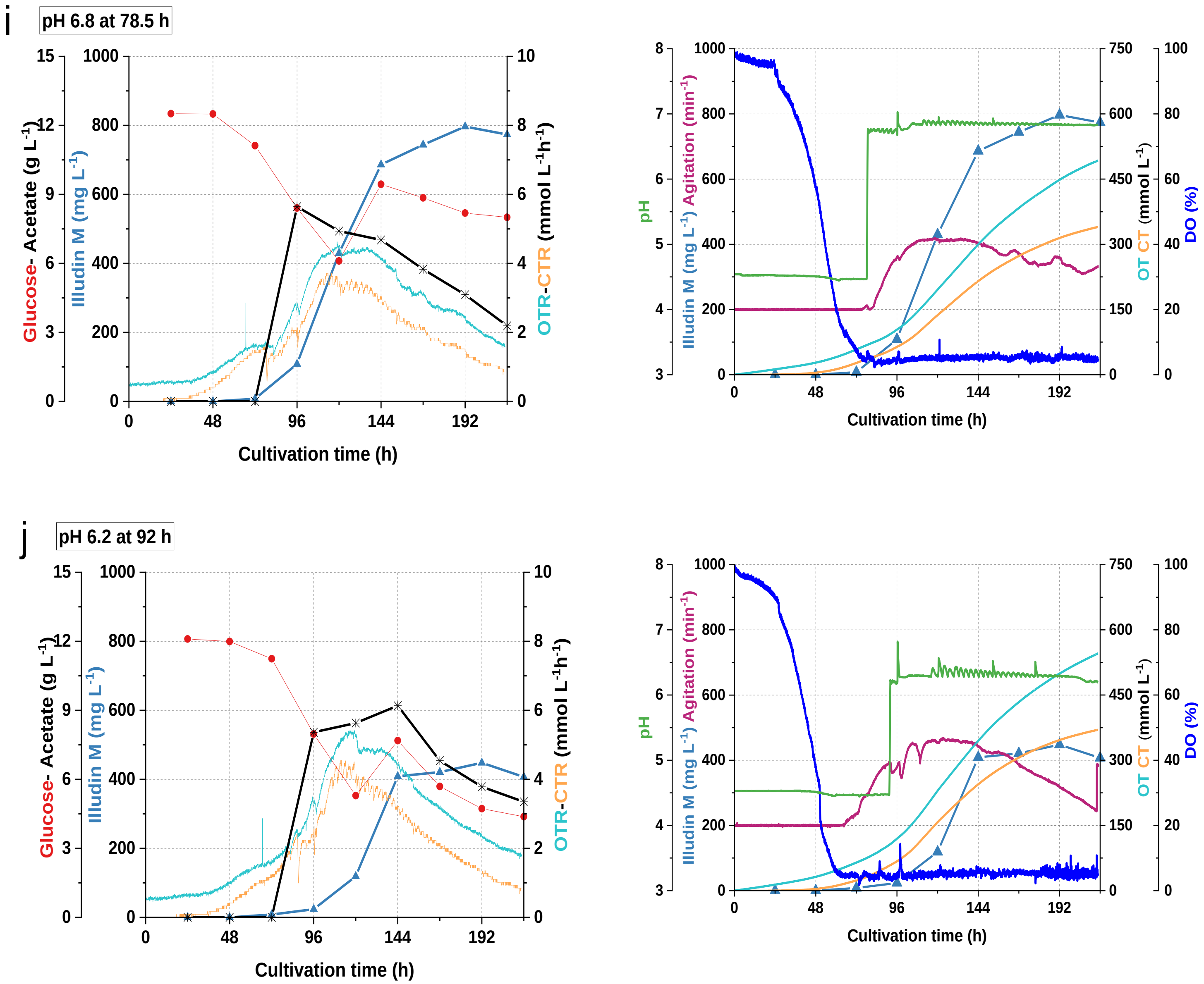

Fig. S 4 Process kinetics from 1.5 L cultivations in stirred tanks for the screening of different pH values and pH shift times. All cultures were prepared with *O. nidiformis* cultivated in G13.5/C7 medium at 23°C, 0.3 vvm (27 sL h^{-1}) aeration and dissolved oxygen (DO) was maintained at 5% by increasing the stirring speed. Acetate was fed at 96 h (8 g L^{-1}), 120 h (4 g L^{-1}) and 144 h (4 g L^{-1}). A feed of glucose was performed at 120 h (6 g L^{-1}). All curves illustrating the course of the different process parameters are colored according to the colors of the axis labels. **i** pH shifted to 6.8 at 78.5 h. **j** pH shifted to 6.2 at 92 h. Illudin M titers were derived from cell free culture supernatant.

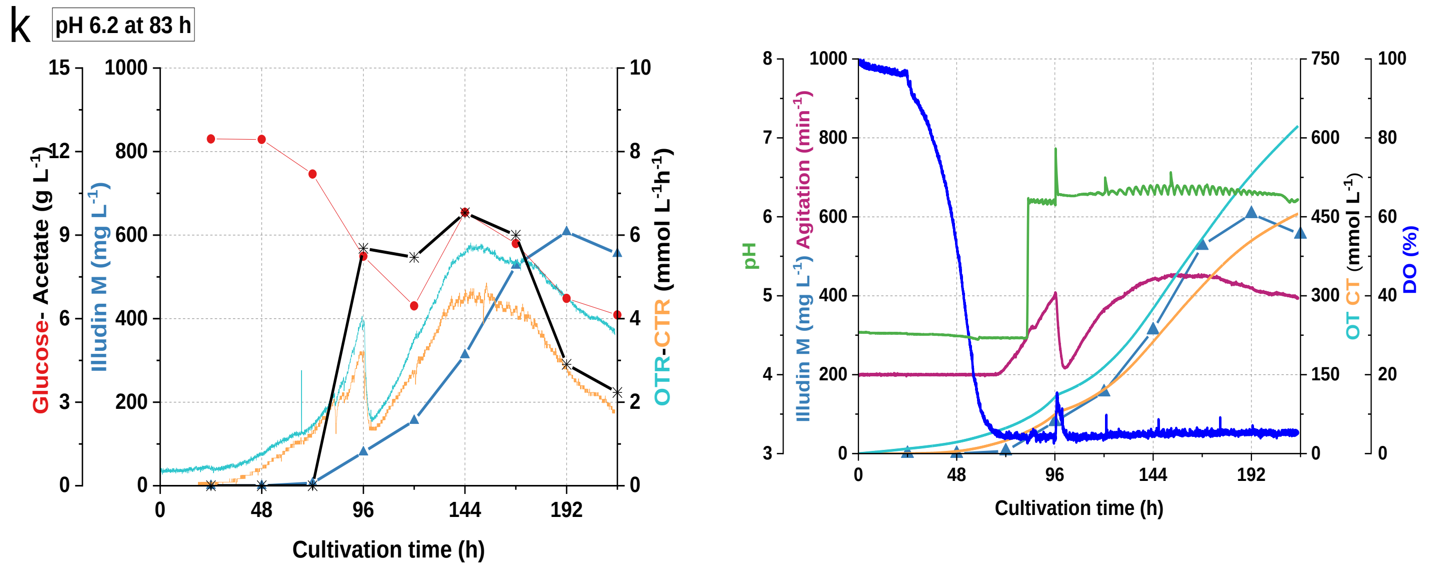

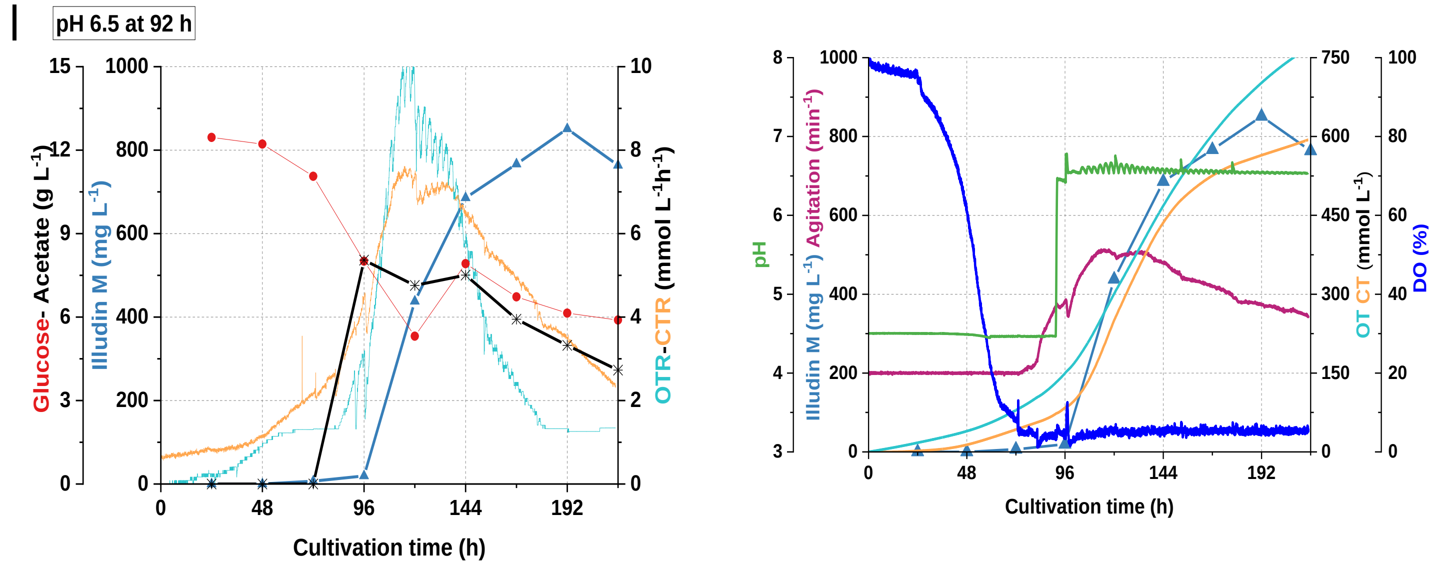

Fig. S 4 Process kinetics from 1.5 L cultivations in stirred tanks for the screening of different pH values and pH shift times. All cultures were prepared with *O. nidiformis* cultivated in G13.5/C7 medium at 23°C, 0.3 vvm (27 sL h^{-1}) aeration and dissolved oxygen (DO) was maintained at 5% by increasing the stirring speed. Acetate was fed at 96 h (8 g L^{-1}), 120 h (4 g L^{-1}) and 144 h (4 g L^{-1}). A feed of glucose was performed at 120 h (6 g L^{-1}). All curves illustrating the course of the different process parameters are colored according to the colors of the axis labels. **k** pH shifted to 6.2 at 83 h. **l** pH shifted to 6.5 at 92 h. Illudin M titers were derived from cell free culture supernatant.

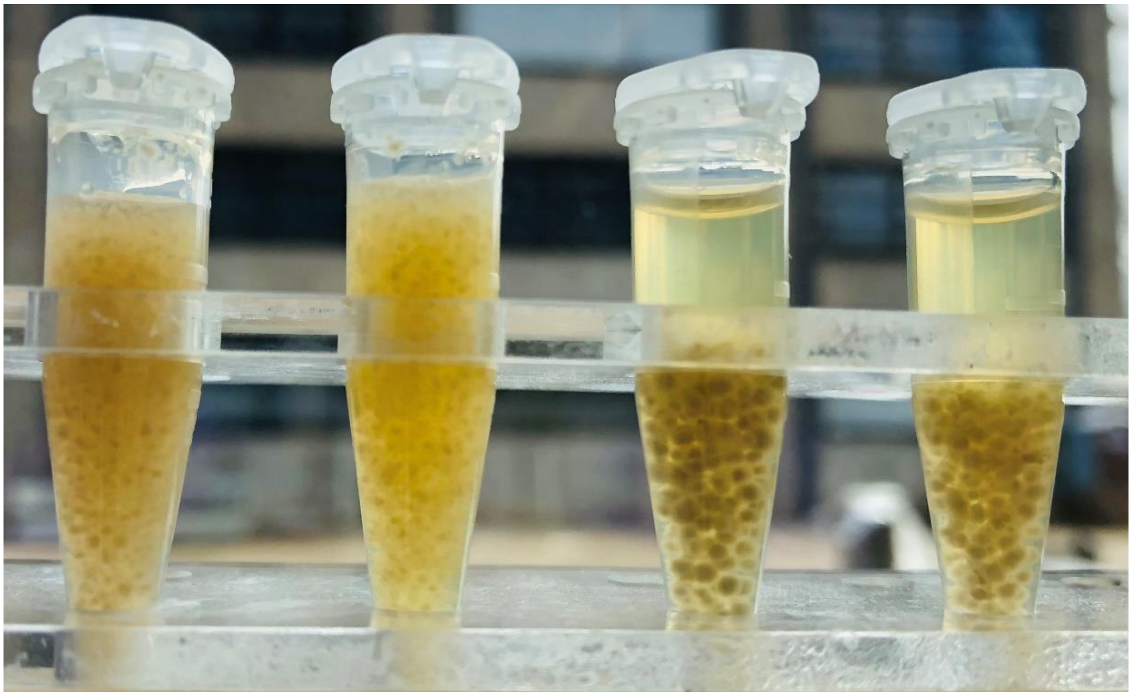

Fig. S 5 Appearance of pellets in samples of submerged cultivations of *Omphalutus nidiformis*. The two tubes on the left contain samples from the best stirred tank cultivation (1.5 L) and the tubes on the right, samples from the optimized shake flask cultivation that was performed in parallel. Both cultivations were inoculated with the same inoculum. Samples were taken at 192 h of cultivation. Picture was taken after 20 min of standing to let the pellets settle in the tubes.

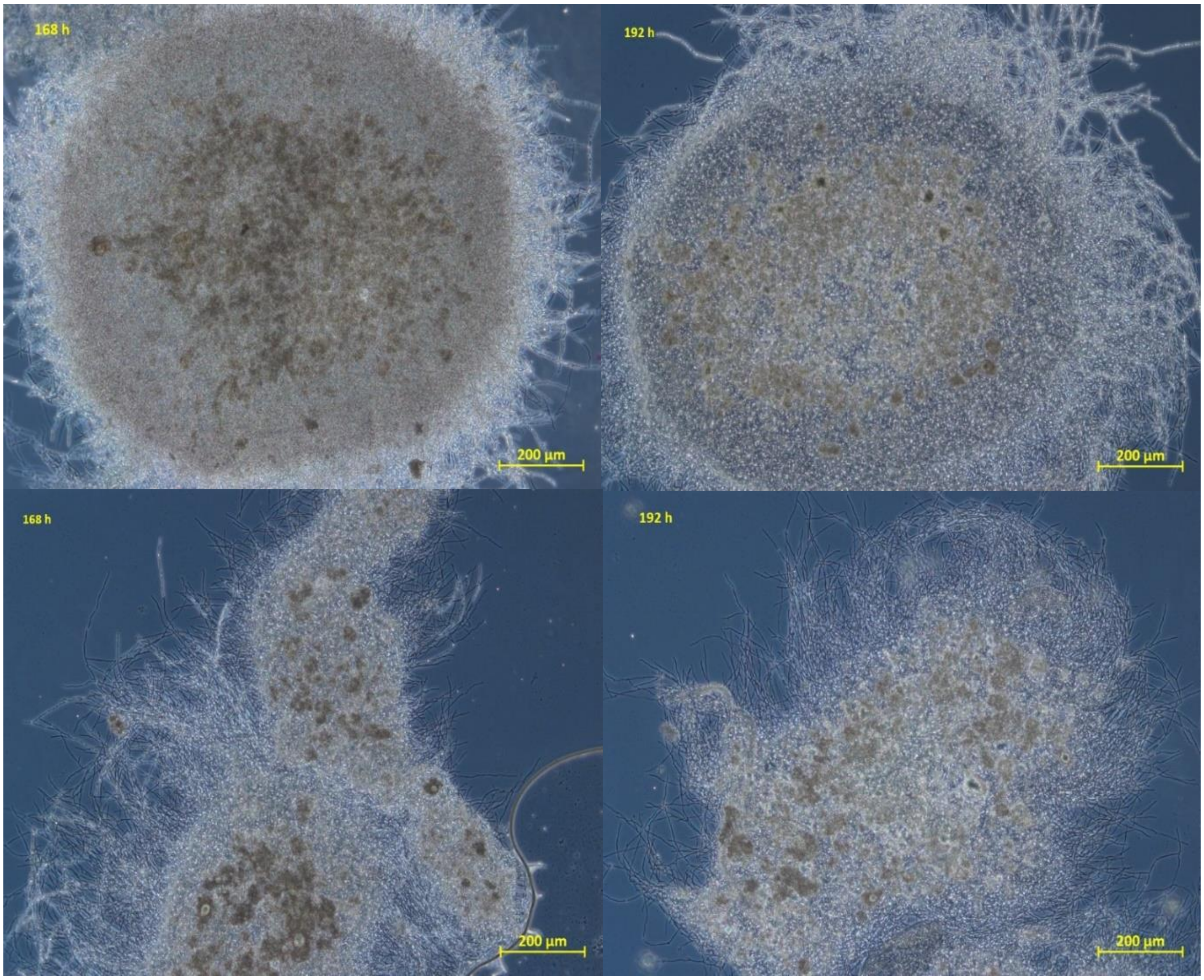

Fig. S 6 Microscopic appearance of pellets from submerged cultivations of *Omphalutus nidiformis.* The upper row shows pellets from an optimized shake flask cultivation, and lower row shows pellets from the best bioreactor cultivation. Microscope was set to 10X magnification for all pictures.

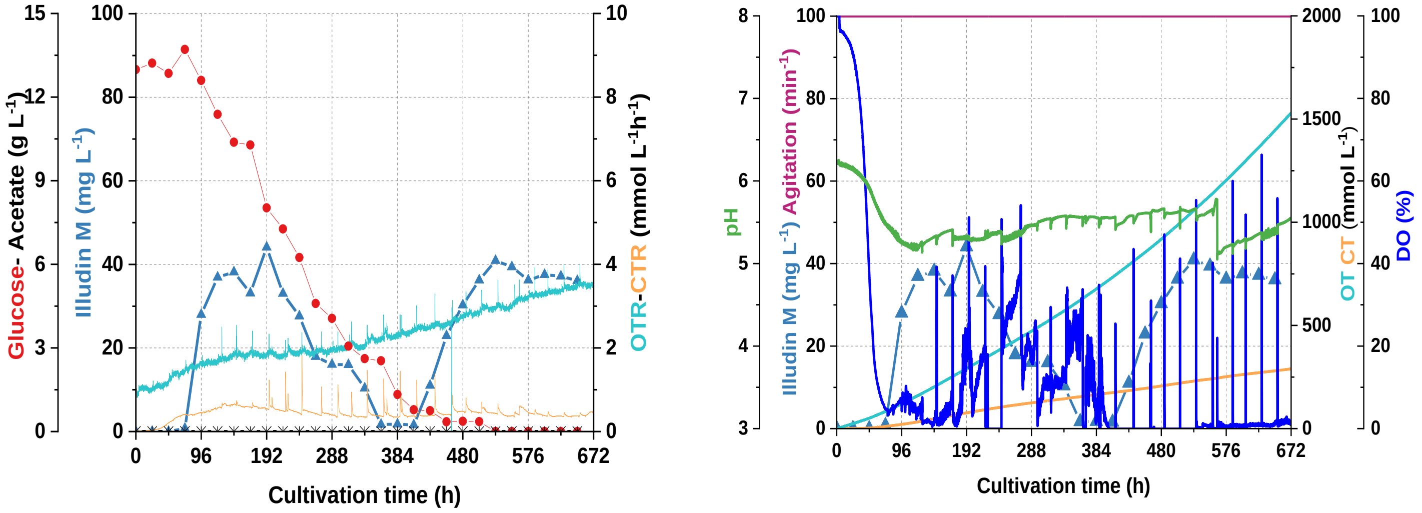

Fig. S 7 Process kinetics from a non-optimized 10 L cultivation in a stirred tank for the production of illudin M . The culture was prepared with *O. nidiformis* cultivated in G20/C5 medium at 23°C, 0.1 vvm aeration, dissolved oxygen (DO) and pH were not controlled.
